# Broodmate aggression and life history variation in accipitrid birds of prey

**DOI:** 10.1002/ece3.5466

**Published:** 2019-07-23

**Authors:** Tomás Redondo, José María Romero, Ricardo Díaz‐Delgado, Jenő Nagy

**Affiliations:** ^1^ Estación Biológica de Doñana CSIC Sevilla Spain; ^2^ Department of Evolutionary Zoology and Human Biology University of Debrecen Debrecen Hungary

**Keywords:** Accipitriformes, life history, phylogenetic generalized least squares, siblicide, sibling aggression

## Abstract

Aggressive sibling competition for parental food resources is relatively infrequent in animals but highly prevalent and extreme among certain bird families, particularly accipitrid raptors (Accipitriformes). Intense broodmate aggression within this group is associated with a suite of traits including a large adult size, small broods, low provisioning rates, and slow development. In this study, we apply phylogenetic comparative analyses to assess the relative importance of several behavioral, morphological, life history, and ecological variables as predictors of the intensity of broodmate aggression in 65 species of accipitrid raptors. We show that intensity of aggression increases in species with lower parental effort (small clutch size and low provisioning rates), while size effects (adult body mass and length of nestling period) are unimportant. Intense aggression is more closely related to a slow life history pace (high adult survival coupled with a restrained parental effort), rather than a by‐product of allometry or food limitation. Consideration of several ecological variables affecting prey abundance and availability reveals that certain lifestyles (e.g., breeding in aseasonal habitats or hunting for more agile prey) may slow a species’ life history pace and favor the evolution of intense broodmate aggression.

## 1 INTRODUCTION

In a diverse minority of bird taxa, nestlings aggressively compete with their broodmates for food, often causing their death (siblicide) due to physical lesions, starvation, or eviction (Mock, Drummond, & Stinson, 1990; Mock & Parker, 1997). Nestling aggression and lethal resource monopolization are rare or absent in most avian families, but highly prevalent among some large, long‐lived carnivorous birds such as boobies, herons, pelicans, and raptors (Drummond, 2002; Mock et al., 1990; Mock & Parker, 1997). This variation has puzzled evolutionary ecologists for decades (Mock, 2004), but the reasons underlying it still remain obscure (Drummond, 2002, 2006; Mock & Parker, 1997).

Most hypotheses proposed to explain interspecific variation in avian broodmate aggression are framed in terms of cost‐effectiveness (Mock & Parker, 1997). Assuming that aggressive rivalry entails direct individual costs to aggressors (energy, risk, and lost opportunity, Lamey & Mock, 1991), aggression will become profitable only in certain species having some traits during the nestling period that make aggression costs to be compensated for. Seven such aggression‐promoting traits have been hypothesized:

1. Monopolizable food items, that is, when small food parcels are passed directly from parent's to chicks' mouths (the “feeding method” or “prey size hypothesis”; Mock, 1985; but see González‐Voyer & Drummond, 2007).
2. Small brood size. The benefits of aggression (the per capita share of food gained by dominant chicks, O'Connor, 1978; Godfray & Harper, 1990; Mock & Forbes, 1994; Mock & Parker, 1997) and its costs (difficulty and risk of establishing and maintaining aggressive dominance relationships among broodmates, Stinson, 1979; Drummond, 2001, 2002) decrease and increase, respectively, with increasing brood size.
3. Parents feeding their chicks with large and infrequent food parcels clustered in bouts or meals, because aggression may be more profitable when it yields a higher net benefit of food reward for the aggressor (the “food parcel size” hypothesis; Drummond, 2002).
4. Aggressive potential (sufficient maturity and size at early nestling ages; Drummond, 2002).
5. Slow food transfer (there is sufficient time between parental arrival and food transfer for nestlings to intervene aggressively; Drummond, 2002).
6. Potential to influence subsequent competitiveness (there are prospective benefits of establishing an aggressive dominance hierarchy among broodmates; Drummond, 2002).
7. Long nestling periods, because investment in establishing a costly dominance hierarchy is more likely to be compensated when broodmates cohabit and compete for a long period, and long nestling periods give more opportunity for serious food scarcity to arise (González‐Voyer, Székely, & Drummond, 2007).

In the single comparative study performed up to date, comprising 69 species in seven bird families, González‐Voyer et al. (2007) found that both the intraspecific prevalence and the intensity of aggression increased with long fledging periods and (contrary to predictions) indirect feeding. A small clutch size (as a proxy of brood size at hatching) was also associated with intense aggression, but neither daily feeding rate (as a proxy of food parcel size) nor egg mass (as a proxy for hatchling mass) was correlated with either the incidence or intensity of aggression.

Diurnal birds of prey in the order Accipitriformes (eagles, hawks, ospreys, and vultures) comprise the largest taxon among bird families where broodmate aggression is both prevalent and intense, but a high degree of behavioral diversity still exists within this group. In some species (e.g., Verreaux's *Aquila verreauxii* and crowned eagles *Stephanoaetus coronatus* or bearded vultures *Gypaetus barbatus*), senior nestlings almost invariably kill their younger broodmates soon after hatching (Brown, 1990; Brown & Amadon, 1968). In others, aggression and siblicide may or may not occur depending on environmental conditions (e.g., food limitation), as in goshawks *Accipiter gentilis* (Squires & Reynolds, 1997) or ospreys *Pandion haliaetus* (Machmer, 1992; Poole, 1982). In contrast, other species of hawks, harriers, and kites, show few signs of aggression despite sharing many traits with their more aggressive relatives (e.g., resource shortages, monopolizable prey items, weaponry, crowded nest conditions, and asynchronous hatching, Balfour, 1957; Newton, 1979; Mock & Parker, 1997). Moreover, this remarkable degree of interspecific variation in broodmate aggression has long been recognized to be associated with other traits linked to a species’ life history such as body mass and clutch size (Mock & Parker, 1997; Newton, 1977; O'Connor, 1978; Simmons, 1988; Stinson, 1979). All these features make accipitrid raptors a promising model system for testing alternative ideas on the evolution of nestling aggression in birds.

In Accipitrids, sibling aggression shows a continuum where larger species display more frequent and sustained aggression, lay smaller clutches, take longer to grow (Newton, 1977), and feed their nestlings at lower rates (Bortolotti, 1986a; Stinson, 1979). This provides several alternative evolutionary explanations for the occurrence of broodmate aggression. For example, according to cost‐effectiveness hypotheses, certain combinations of these correlated traits (e.g., a small brood size and long nestling period) make aggression profitable at the nestling stage in some species but not in others (Drummond, 2002; Mock & Parker, 1997). Alternatively, all these traits (including broodmate aggression) may be the coadapted, evolutionary outcome of selective pressures acting at an older life stage. For example, Simmons (1988, 1991) observed that more aggressive (and particularly obligate siblicidal) species of raptors: (a) were larger; (b) laid smaller clutches; (c) showed delayed acquisition of adult plumage; and (d) lived mainly in tropical habitats. He suggested that this syndrome of traits indicated that aggressive species were long‐lived (e.g., Bennett & Owens, 2002) and, therefore, suffered from high subadult mortality and intense competition for breeding sites. He concluded that broodmate aggression in these species was the end result of selection for offspring quality (growing faster and fledging heavier, thus enhancing juvenile survival) and competitive ability (domination of subordinate siblings) in order to increase the chances of winning a breeding opportunity (Simmons, 1988, 1991). In this case, broodmate aggression and the other traits may correlate because they are caused by selection acting on a different trait (e.g., age‐specific mortality). Finally, a third possibility is that broodmate aggression is directly caused by a large body mass, and this explains why it correlates with the remaining life‐history traits. For example, the importance of existence energy costs to the total energy requirements of nestlings decreases with increasing body mass. For large birds such as eagles, growth would be detrimentally affected if food becomes limited (Bortolotti, 1986a), but the young may receive enough energy to satisfy their relatively smaller existence energy requirements. Therefore, relatively large species may more frequently employ violent sibling aggression than do small species, in order to cause a drastic reduction in food intake below the level required for a competing sibling to survive (Bortolotti, 1986b).

Unraveling the evolutionary causes of broodmate aggression requires using comparative methods to determine the relative contribution of these variables to explain the observed variation between species (González‐Voyer et al., 2007). Comparative methods, however, only provide correlations and do not distinguish cause and effect (Harvey & Pagel, 1991; Partridge & Harvey, 1988), which makes it difficult to discriminate within a set of candidate predictors that correlate with each other. One possible solution to distinguish among these alternative causal models is to apply confirmatory path analysis, a type of structural equations modeling that not only minimizes the confounding effect of collinearity, but actually exploits correlations between predictors to infer both direct and indirect relationships (González‐Voyer & von Hardenberg, 2014; von Hardenberg & González‐Voyer, 2013; Maness & Anderson, 2013). In this study, we use phylogenetic comparative methods to, first, determine which among this set of correlated traits best predicts the intensity of broodmate aggression in accipitrids. Second, we clarify the causal relationships among these traits by testing three alternative evolutionary scenarios: (a) a low nest provisioning rate is the ultimate cause of nestling aggression, slow development, and low fecundity (the *Provisioning* scenario, Lack, 1968; Sæther, 1994); (b) body mass is the key variable determining fecundity, provisioning, and development rates (the *Allometry* scenario, Western & Ssemakula, 1982; Calder, 1984); and (c) low reproductive effort (clutch size) is the principal causal force of variation in the other traits (the *Fecundity* scenario, Charlesworth, 1994; Ricklefs, 2000).

In addition to the above axes of life history variation (size and reproductive effort), we explore how different lifestyles affecting the ease of resource acquisition (Sibly & Brown, 2007; Sibly et al., 2012) may explain variations in the intensity of broodmate aggression. Specifically, we test the predictive value of some ecological factors likely to affect prey abundance and availability. For example, in less productive or aseasonal habitats, where food resources are more limited during the breeding season, parents may feed their nestlings with food parcels which are either scarce or unpredictable, and aggressive competition among siblings may ensue (Poole, 1982). Also, food niche breadth may affect provisioning patterns, with generalist/opportunistic hunters experiencing higher or more predictable feeding rates (Newton, 1977, 1979) or, alternatively, a lower hunting efficiency (Terraube, Arroyo, Madders, & Mougeot, 2011). Species hunting for larger or warm‐blooded prey (mammals and birds) may provide nestlings with more biomass, but at lower provisioning rates (Newton, 1977, 1979; Sæther, 1994). Provisioning rates of nestlings also depend on both hunting effort and success, and the latter may vary widely according to prey type. Among raptors, hunting success is highest in those species preying on relatively small, easily dispatched prey such as invertebrates or herpetofauna and lowest among species hunting for relatively large, agile prey, particularly birds (Temeles, 1985; Toland, 1986). No previous study has so far attempted to explore such plausible relationships between interspecific variations in the intensity of broodmate aggression and foraging lifestyles for any group of birds.

## 2 METHODS

### 2.1 Broodmate aggression, behavior, and life history traits

**Figure 1:**
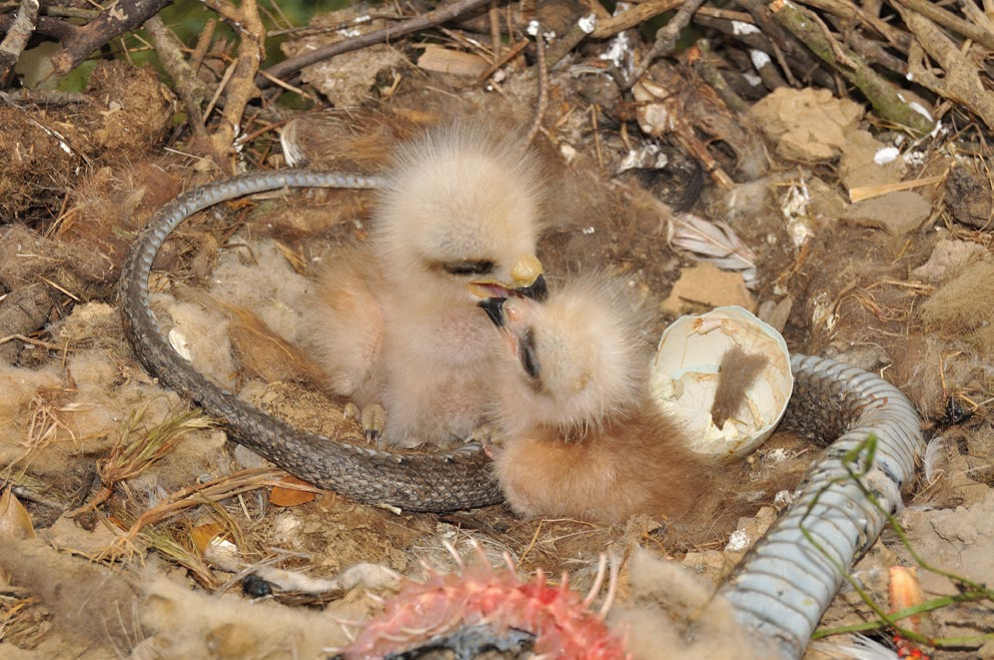
A just‐hatched Black kite *Milvus migrans* nestling is attacked (shaked and pecked on the head) by its older and larger sibling (Photo credit: F. Sergio)

Data on behavioral (intensity of aggression, nestling provisioning rates, feeding method, hunting success, and migration) and life history traits (body mass, clutch size, and length of nestling period) were collected from publications and reference books for 65 species from 26 genera of accipitrid raptors, which represent more than one‐third of all genera of Accipitriformes (BirdLife International, 2015). Intensity of aggression was measured as in González‐Voyer et al. (2007) on a 4‐point scale: 0 (no aggression observed), 1 (few fights or few pecks per fight), 2 (an intermediate number of fights or pecks), and 3 (common and/or long fights, severe injuries, or fratricide; Figure 1). Nestling provisioning rate was measured as the number of feeding trips or prey items carried to the nest per hour during the nestling period. Data on hourly feeding rates were collected for 35 out of 65 species. When the information available consisted of daily feeding rates, we divided by the average day length (in hours; González‐Voyer et al., 2007). In 15 of these cases, information about the exact dates or locations of field studies could not be assigned unambiguously. Hence, we assumed a daylight duration of 12 hr for tropical and 14 hr for temperate (above 23° latitude) species. When data were given for different nestling ages, those closest to the middle of the nestling period were chosen. The average value was computed when data from several sources were available. Following González‐Voyer et al. (2007), feeding method was measured as the fraction of nestling period during which feeding is direct (i.e., beak‐to‐beak), ranging from 0 (indirect feeding throughout the nestling period) to 1 (direct feeding throughout the nestling period). For species with a developmental transition in feeding method, the fraction was computed on the basis of the average age at which chicks switched from one method to the other. Data on feeding method were available for 57 species. Most raptor species defend large breeding territories and thus typically occur at very low local densities. As a result, estimates of behavioral variables are sometimes based on small sample sizes, which may raise questions about data quality and limitations. For example, an aggression level of 0 could mean either that this species nestlings really do not attack one another or that the field worker(s) did not sample a sufficient number of nests or spend enough time on watching to detect it. We collected data on sampling effort for intensity of broodmate aggression and its two behavioral predictors (provisioning rate and feeding method) as the minimum number of broods observed to estimate them which were reported in bibliographic sources and references therein. When the exact number of broods could not be determined, we assumed a minimum number of one brood per bibliographic source.

Mean values of clutch size were collected, or modal values were used in cases where the former were not available. Body mass (in grams) was collected as the average for both sexes. Length of nestling period was collected as the period in days during which nestlings remain in the nest before abandoning it definitely. In most accipitrid raptors, the length of nestling period is equivalent to fledging time (the period necessary for nestlings to complete their postnatal development, acquire their juvenile plumage, and perform their first flights), with a few exceptions. For example, nestlings of hen harrier (*Circus cyaneus*) take three weeks to fledge, but become ambulatory when a. 2‐week old, moving into the surrounding vegetation, so they are no longer confined to the nest during feeding sessions at the last third of the fledging period (Smith, Wittenberg, Macwhirter, & Bildstein, 2011). Based on the data in the monography of Ferguson‐Lees and Christie (2001), migratory behavior was coded as two categories: (0) sedentary and (1) migrant, including partially migrant species in which only part of the population is migratory and obligatory migrant species with clearly distinguishable breeding and wintering areas (Nagy, Végvári, & Varga, 2017).

### 2.2 Diet and prey types

Diets were categorized into nine prey classes ranked by importance: (1) bird, (2) mammal, (3) reptile, (4) fish, (5) amphibian, (6) crustacean, (7) insect, (8) worm, and (9) carrion, and diet breadth and reliance on warm‐blooded prey were calculated following Roulin and Wink (2004), Nagy and Tökölyi (2014), and Nagy et al. (2017). For example, the Golden Eagle (*Aquila chrysaetos*) feeds mostly on mammals and birds, commonly reptiles, occasionally amphibians and fish, even insects and carrion (Ferguson‐Lees & Christie, 2001); thus, its dietary breadth counted as seven, calculated as the number of the listed categories. The average score of mammals and birds was computed as the level of reliance on warm‐blooded prey (ranked between 1 and 9, where lower values mean higher dependency). In this example, the two values are 1 and 2 for mammals and birds, respectively, thus the level of reliance on warm‐blooded prey is 1.5 for this species. A score of prey agility was calculated by the weighted mean of consumed prey types using a 5‐point scale: 1 (almost or completely immobile, carrion and worms), 2 (somehow mobile, insects and crustaceans), 3 (mobile with bad maneuverability, reptiles and amphibians), 4 (agile, fish and mammals), and 5 (highly agile, birds). As a measure of the difficulty in capturing prey, we also collected data on hunting success for 40 out of the 65 species included in the dataset. Hunting success was measured as the fraction of successful attacks on prey obtained from published field observational studies. Hunting success for the two vulture species was assumed to be maximum (0.9).

### 2.3 Ecosystem functionality and distribution

Several procedures have been proposed to estimate ecosystem primary production from remote sensing images. Among them, MODIS Gross and Net Primary Productivity products (GPP/NPP MOD17) have been found to be a close surrogate for most of the terrestrial biomes (Turner et al., 2006; Zhang, Xu, Chen, & Adams, 2009). Based on the theory suggested by Monteith (1972, 1977), GPP and NPP under nonstressed conditions are linearly related to the amount of Absorbed Photosynthetic Active Radiation (APAR), which can be inferred from spectral vegetation indices (visible and infrared combination of sensor bands). Thus, MOD17 GPP provides an accurate estimate of the amount of biomass that plants create in a given length of time, from which a fraction is used for respiration, while NPP will be the remnant fraction which is fixed by vegetation. Throughout a growing cycle, the global MOD17 GPP/NPP products depict the phenological changes in relation to carbon uptake and vegetation growth. The global extent and broad scale of these scientific products make them suitable to investigate the different GPP/NPP ranges hosting bird populations. There are two caveats with respect to the usability of MOD17 time series: cloud contamination of image composites and spatial resolution inconsistencies with the ancillary meteorological gridded data. Accordingly, we selected the MOD17A3‐improved dataset processed by Zhao, Heinsch, Nemani, and Running (2005) which solved both constraints. This product is provided in monthly composites for GPP and on a yearly basis for NPP (Huertas, Peri, Diaz‐Delgado, & Martínez Pastur, 2016; Martínez Pastur et al., 2018). The time series used in this study spans from 2000 to 2014.

Prior to GPP extraction, bird distribution areas (BirdLife International, 2015) were refined according to altitudinal ranges for each species obtained from the literature. Mean GPP was then extracted for every refined bird distribution area producing 180 monthly values per species covering the 15‐year GPP times series. Since we were mainly interested in how habitat productivity affected food abundance during the chick‐rearing period, for migratory species, values were computed for breeding areas only. We defined the breeding season of a species as the number of months ranging from clutch initiation to chick rearing, obtained from the literature. When a species had two breeding seasons in the same area, bred all year round, or bred at both winter and nonwinter quarters, all months when breeding occurred were considered for computing breeding GPP variables.

We computed several derived variables from this 15‐year GPP dataset related to habitat productivity and stability. First, we computed average GPP of all 180 months (mean GPP), the least productive (min GPP) and the most productive (max GPP) month (i.e., the minimum and the maximum of all values during the 15 years, respectively) to measure general patterns of environmental productivity throughout the whole period. Furthermore, average GPP exclusively for the breeding season was also calculated, only including monthly values of species‐specific length of season in which breeding occurs (mean GPP breeding). Second, for measuring within‐year habitat stability (seasonality), we computed the annual range as the difference (max GPP‐min GPP) averaged across 15 years (annual range GPP) and the averaged standard deviation of GPP values per year (annual *SD* GPP). Third, measures of between‐year variation in GPP values were calculated as the standard deviation of the least productive months (*SD* of min GPP), and the standard deviation of the most productive months (*SD* of max GPP). Similarly, these values were also computed for all months when breeding occurred (*SD* of GPP breeding), as well as the total measure of the standard deviation of all 180 months (*SD* GPP, all values).

There was a huge variation in the surface areas of distribution ranges among the studied species (range 77–3,567,456 km^2^). This might potentially bias estimates of variation in GPP values, because species inhabiting large areas may account for larger local differences in rainfall and GPP, particularly in tropical species with extended breeding seasons. Thus, we checked that no positive correlations existed between total area of distribution range and either duration of the breeding season (Spearman's correlation, *r*_s_ = .13, *p* = .319), mean GPP (mean GPP: *r*_s_ = −.22, *p* = .075; mean GPP breeding: *r*_s_ = −.16, *p* = .189; annual range GPP: *r*_s_ = −.11, *p* = .369; min GPP: *r*_s_ = −.15, *p* = .240; max GPP: *r*_s_ = −.18, *p* = .140) or *SD* values (*SD* GPP: *r*_s_ = −.11, *p* = .404; *SD* of GPP breeding: *r*_s_ = −.05, *p* = .703; annual *SD* GPP: *r*_s_ = −.10, *p* = .439). A negative correlation existed between total area of distribution and both *SD* of min GPP (*r*_s_ = −.25, *p* = .048) and *SD* of max GPP (*r*_s_ = −.31, *p* = .013).

Length of the breeding season (in months), together with maximum altitude (m) in the breeding areas, and migration behavior were considered as variables of spatial distribution in this study.

### 2.4 Comparative analyses and phylogeny

**Figure 2:**
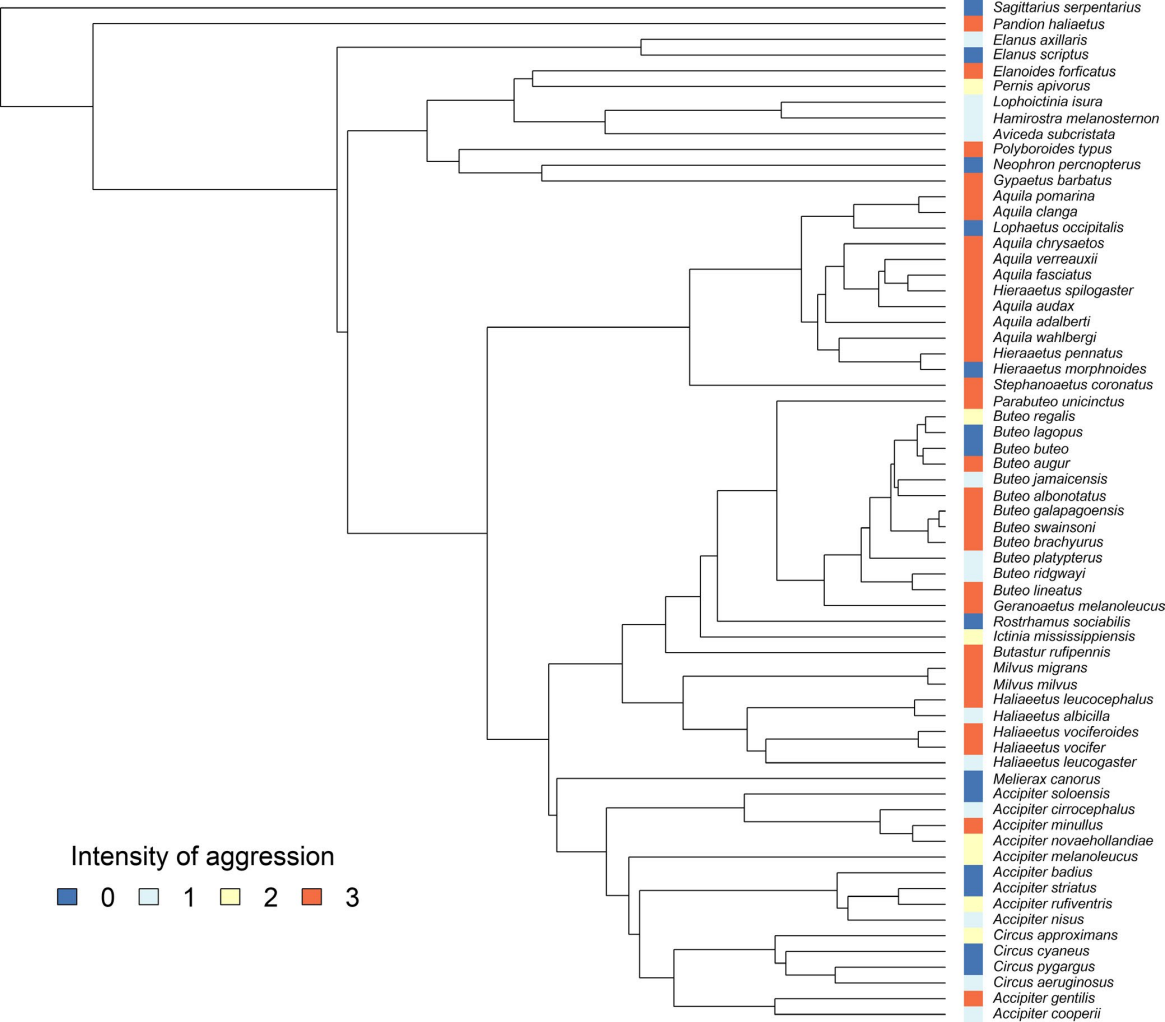
Phylogenetic tree of species used in the study and phylogenetic distribution of the intensity of broodmate aggression (in a color scale)

We used phylogenetic comparative analyses (Paradis, 2014) to test for correlations between the intensity of aggressive competition by broodmates and life history traits (body mass, clutch size, and nestling period) and parental feeding behavior (feeding method and provisioning rate), as well as ecological factors likely to affect food abundance and availability. Effects of predictors were estimated using maximum likelihood‐based methods after controlling for the phylogenetic relationships among species, using phylogenetic generalized least squares regression (PGLS; Martins & Hansen, 1997; Symonds & Blomberg, 2014), as implemented in the “ape” and “nlme” R packages (Paradis, Claude, & Strimmer, 2004; Pinheiro, Bates, DebRoy, Sarkar, & R Development Core Team, 2016). This approach allows to include multiple predictors in a single analysis completed with fitting the model of trait evolution. Pagel's *λ* (Pagel, 1997, 1999), that is, the phylogenetic signal, is a quantitative measure of trait relatedness to the phylogeny of species. It can vary from *λ* = 0 (no correlation exists among species with different level of phylogenetic relatedness) to *λ* = 1 (a Brownian motion model, indicating dependency in the evolution of the trait; Kamilar & Cooper, 2013). Values of best‐fitted *λ* were estimated from likelihood‐profiles of the parameter (Kamilar & Cooper, 2013) including 500 randomly selected values between 0 and 1, dynamically changed in each PGLS run. The *λ* value with the lowest log likelihood is reported for each model. A molecular phylogenetic tree based on ten (nuclear and mitochondrial) genes was used (Nagy & Tökölyi, 2014). This tree was modified by manually adding six taxa (namely: *Circus approximans*, *Accipiter badius*, *Accipiter melanoleucus*, *Accipiter minullus*, *Elanus scriptus*, and *Elanus axillaris*) based on a consensus tree of random phylogenies downloaded from Birdtree.org (Jetz, Thomas, Joy, Hartmann, & Mooers, 2012), after further verification of the phylogenetic position of the species in previously published studies (Oatley, Simmons, & Fuchs, 2015; Roulin & Wink, 2004; Wink & Sauer‐Gürth, 2004; Figure 2). The effect of tree topology on the analyses was checked in a preliminary phase, resulting in no differences in the outputs, thus our own‐built tree was used in all cases.

### 2.5 Model selection

We performed an Information Theory (IT)‐based PGLS model selection procedure and multimodel inference (Burnham & Anderson, 2002; Garamszegi & Mundry, 2014; Grueber, Nakagawa, Laws, & Jamieson, 2011) to determine which predictors had the strongest effect on the intensity of broodmate aggression. First, we identified the most important behavioral and life history predictors among those suggested by previous studies to directly cause nestling aggression in raptors and other birds (adult body mass (Bortolotti, 1986a; Simmons, 1988); provisioning rate (Drummond, 2002); clutch size (Drummond, 2002; Mock & Parker, 1997); feeding method (Mock & Parker, 1997); and length of nestling period (González‐Voyer et al., 2007) using the dataset containing 57 species. We established an a priori candidate set of 25 different combinations of 1–4 predictors plus a null model containing no predictors at all. Body mass and length of nestling period were highly correlated (Spearman *r*_s_ = .84, *p* < .001), and this collinearity might bias multimodel inference in several ways (Freckleton, 2011). Therefore, we excluded from the “a priori” model set those models where body mass and nestling period entered simultaneously together with other predictors. This kept variance inflation factors below 2.5, which is acceptable (Zuur, Ieno, & Elphick, 2010). This analysis was repeated using a broader dataset of 65 species without feeding method as a predictor.

Second, we performed a model selection procedure to determine which ecological variables, related to diet, location, habitat productivity, and stability, were important predictors of broodmate aggression. As a first step, we selected those variables which explained a substantial amount of variance in either aggression or its main life history predictors found in the previous analysis (provisioning rate, clutch size, and feeding method) by performing simple PGLS runs. Some ecological variables were highly correlated and provided redundant information. Thus, for each group of highly correlated ecological predictors, we selected only the variable with the best predictive value for the intensity of broodmate aggression based on AICc values (see below). Then, we established an a priori candidate set of different combinations (all‐subset selection) of nine selected ecological predictors of the intensity of broodmate aggression by using the permutations function in the “gtools” R package (Warnes, Bolker, & Lumley, 2018). This procedure is considered a sensible method when testing causal relationships between explanatory variables and the response variable (Harrison et al., 2018). In a second step, we repeated the same analysis by adding the two most important life history predictors (provisioning rate and clutch size) to verify whether ecological variables could still explain variations in the intensity of broodmate aggression even when variations in life history were taken into account.

The model selection procedure involved ranking all models by their Akaike information criterion corrected for small samples (AICc) and selecting a top subset of plausible models within ΔAICc < 10 from the top model (Burnham & Anderson, 2002; Symonds & Moussalli, 2011). We used the “AICcmodavg” R package (Mazerolle, 2017). Following Anderson (2008) and Arnold (2010), we checked whether some of the models in the top subset were simply more complex versions of nested models with better AIC support, in order to remove them from the top subset and recalculated model AICc weights considering only these truly competing models. Although we avoided building models containing combinations of predictors showing a strong collinearity (e.g., body mass and nestling period), even moderate amounts of collinearity may strongly affect IT‐based multimodel inference based on model averaging (Cade, 2015). Therefore, variable importance was computed as ratios of standardized regression estimates obtained by model averaging (Burnham & Anderson, 2002) of the best subset of truly competing models with ΔAICc < 10, weighted by its recalculated Akaike weight. Parameter estimates were computed by averaging all models in the best subset, substituting estimates (and error) by zero into those models where the given parameter was absent (Burnham & Anderson, 2002). Standardized estimates of regression coefficients based on partial standard deviations incorporating variance inflation factors were computed according to Cade (2015). Variance inflation factors for predictors in PGLS models were calculated with the functions in the “car” R package (Fox & Weisberg, 2011).

Variables correlating with body mass (clutch size, length of nestling period, and provisioning rate) were positively skewed and were log transformed. Prior to transformation, hourly provisioning rates were converted into circadian rates after multiplying by 24. We also log transformed ecological predictors having a significant positive skewness according to D'Agostino test as implemented in “moments” R package (Komsta & Novomestky, 2015). A logit transformation was used for proportions (feeding method where 1.0 values were converted into 0.95‐ and hunting success; Warton & Hui, 2011). Variables were Z‐transformed (mean centered with *SD* of 1) prior to analyses in order to improve the stability of models and likelihood of model convergence, and the accuracy of parameter estimates (Harrison et al., 2018). All analyses were carried out in R v3.5.1 (R Development Core Team, 2018).

### 2.6 Potential biases caused by heterogeneity in sampling effort

Sample sizes for behavioral variables in the dataset showed considerable variation (range: 1–343 broods; Appendix S1). Such heterogeneity in sampling effort may affect several assumptions of statistical methods and needs to be properly accounted for (Garamszegi, 2014). This is a usual situation in comparative analyses, where there is often an inherent trade‐off among precision and breadth of data (Garamszegi & Møller, 2010). One possible solution to this problem is the exclusion of data for species that do not reach a given threshold. For example, in their comparative study, González‐Voyer et al. (2007) included only species for which a minimum of three broods were observed during at least 5 hr per brood. However, such thresholds may, on their own, introduce additional bias if sampling effort is correlated with some life history (e.g., body mass) or ecological traits (e.g., distributional range; Garamszegi & Møller, 2010, 2012) and raise ethical questions as well (Garamszegi & Møller, 2010). We, therefore, chose including all available data while simultaneously considering differences in precision of estimates of behavioral traits of different species due to differences in sampling effort (Garamszegi, 2014; Garamszegi & Møller, 2010, 2012). In doing so, we assume that the noise in any individual data point is overwhelmed by the broader comparative signal. This assumption was based on three pieces of evidence (Appendix S1; Garamszegi & Møller, 2010, 2012): (a) no bias was detected in estimates of intensity of broodmate aggression according to sampling effort; (b) no evident trend was found in our sample for certain taxa being better studied than others; and (c) models accounting for within‐species variance due to heterogeneity in sampling effort did not offer a better fit to the data than unweighted models.

### 2.7 Confirmatory path analysis

**Figure 3:**
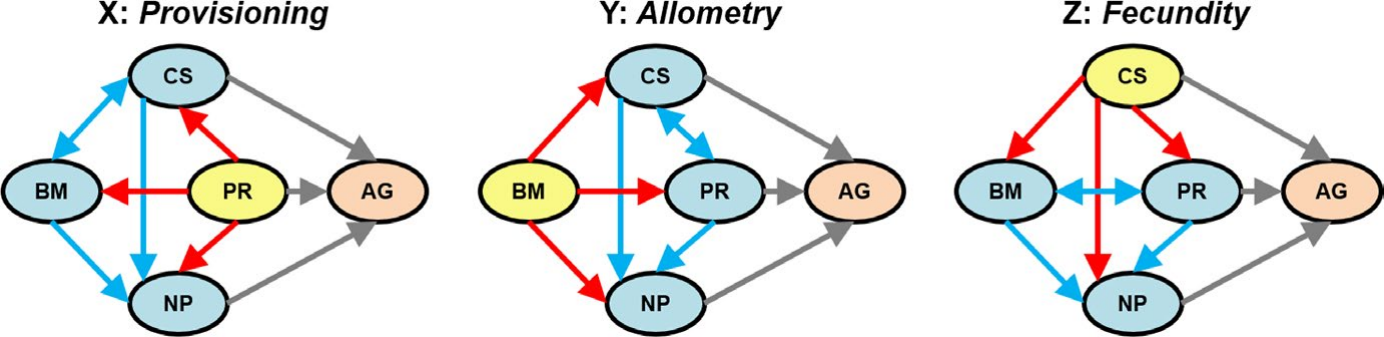
Directed acyclic graphs showing all possible causal relationships between predictors of broodmate aggression (AG) in accipitrid raptors for three alternative evolutionary scenarios defined by the root variable (in yellow), which was assumed to act as the primary cause of all other variables: Provisioning Rate (PR; Layout X, the *Provisioning* scenario), Body Mass (BM; Layout Y, the *Allometry* scenario) or Clutch Size (CS; Layout Z, the *Fecundity* scenario). Each layout is defined by a set of causal relationships classified as Primary (those between the root variable defining each layout and all other variables except Aggression (AG; red arrows), Secondary (those between pairs of variables other than the root variable and AG; blue arrows), and Tertiary (those between variables and AG; gray arrows)

To differentiate between alternative models of direct and indirect paths of causal relationships between broodmate aggression and its associated life history traits, we used phylogenetic path analysis (González‐Voyer & von Hardenberg, 2014; von Hardenberg & González‐Voyer, 2013). We defined a number of possible causal models including intensity of aggression and the four correlated variables (body mass, clutch size, nestling period, and provisioning rate) according to three evolutionary scenarios (Figure 3). The fit of each model was tested applying the d‐separation method (von Hardenberg & González‐Voyer, 2013) using the “cpa” R package (Bellino et al., 2015) modified to perform PGLS instead of linear regressions implemented in the “nlme” package, as described above. The d‐separation method assesses the minimal set of conditional independencies expected for the causal path model to be correct (i.e., fulfilled by the observed data), by estimating Fisher's *C* statistic, a measure of goodness of fit of the model to the data, which can be approximated to a *χ* ^2^ distribution with 2*k* degrees of freedom. A non‐significant *C* statistic indicates that a path model fits the observations, that is, proposed causal relationships are statistically dependent and nonadjacent variables are independent. The fit of different path models can be compared using the *C*‐statistic information criterion (CICc; analogous to the Akaike information criterion; von Hardenberg & González‐Voyer, 2013). We calculated CICc weights for all models, which provide an estimate of the likelihood of each model (Burnham & Anderson, 2002). The model selection procedure in confirmatory path analysis involved selecting a top subset of plausible path models with nonsignificant values of the *C* statistic and then excluding those models which were more complex versions of nested models with better CICc support. Having selected the best subset of truly competing path models, we computed averaged standardized path coefficients (González‐Voyer & von Hardenberg, 2014) after incorporating variance inflation factors according to Cade (2015).

The three alternative evolutionary scenarios considered (Figure 3) propose different causal routes toward the syndrome of traits related to the intensity of broodmate aggression.

#### 2.7.1 Provisioning scenario

A low hunting or provisioning rate (e.g., due to a low abundance or availability of prey) is the ultimate causal factor of broodmate aggression, either directly (chicks fight for infrequent or unpredictable food parcels, Drummond, 2001, 2002) and/or indirectly because food limitation also causes a small clutch size (parents are limited to raise small broods if hunting rates are low, Bortolotti, 1986a; Lack, 1968; Sæther, 1994; Simmons, 2000) and slow development (i.e., a long nestling period, because it takes longer to convert prey biomass into nestling biomass, Lack, 1968). In this scenario, rather than viewing body mass as a fixed constraint, it is allowed to vary in response to selection on other traits (Bennett & Owens, 2002; Partridge & Harvey, 1988). In turn, a large body mass will cause a long nestling period, because larger birds take longer to develop (Starck & Ricklefs, 1998).

#### 2.7.2 Allometry scenario

A large body mass is the indirect, ultimate cause of broodmate aggression, either via a low feeding rate (because large species feed on larger, less abundant prey, Schoener, 1968) or because large body mass also causes a long nestling period and a small clutch size. Since egg production depends on mass‐specific metabolic rate, mass‐specific rate of productivity should scale negatively with body size (Sibly et al., 2012), that is, large species mature later and are less productive (Sibly et al., 2012; Western & Ssemakula, 1982). As in the *Provisioning* Scenario, a low provisioning rate may cause a small clutch size and, conversely, a small brood size may also cause a low provisioning rate due to lower total food demands (Martin, Martin, Olson, Heidinger, & Fontaine, 2000).

#### 2.7.3 Fecundity scenario

A small clutch size is the ultimate cause of broodmate aggression and their correlates (low provisioning rates and long nestling periods). In this scenario, small clutch size is considered as a fecundity “life‐table” variable reflecting life history pace, that is, aggression is ultimately caused by a reduced reproductive effort associated with a low adult mortality rate (Bennett & Owens, 2002; Linden & Møller, 1989). Although annual fecundity (i.e., the product of clutch size and the number of broods per year) is a better predictor of adult mortality rates than clutch size (Bennett & Harvey, 1988; Martin, 1995), most accipitrid raptors produce only one brood per year (Newton, 1979) and, therefore, both variables are highly correlated (Ricklefs, 2000). A small clutch size may directly cause broodmate aggression because it is more efficient in small broods (as in the two previous scenarios), but also because offspring in low‐fertility, long‐lived species may be strongly selected for investing in viability and outcompete broodmates aggressively (Simmons, 1988). A low fecundity (slow life history) may also indirectly affect nestling aggression via a low provisioning rate and a long nestling period. Here, a low provisioning rate is not the result of food limitation (as in the *Provisioning* scenario) but of restrained parental effort (Bókony et al., 2009; Ghalambor & Martin, 2001). Long‐lived species are expected to behave as “prudent parents” during reproduction (Drent & Daan, 1980) in order to not compromise their future survival. Low fecundity may also directly cause long nestling periods, either because a slow life history pace selects for slow development (Metcalfe & Monaghan, 2003; Remeš, 2007) or because low nest attentiveness (including provisioning rates) decrease growth rate and extend the nestling period (Lack, 1968; Martin, 2002; Martin, Oteyza, Mitchell, Potticary, & Lloyd, 2015). As in the *Provisioning* scenario, body mass is allowed to vary in response to selection on the other traits. Again, a large body mass directly causes a long nestling period. Note that body mass can also act as a causal predictor of provisioning rate (as in the *Allometry* scenario; Figure 3).

We made some simplifying assumptions when defining causal path models in order to reduce model space. First, we assumed that Aggression (AG) is never a causal predictor of any other variable (see von Hardenberg and González‐Voyer (2013) for a nonsupported causal effect of broodmate aggression on clutch size), but it may be directly caused by clutch size (CS), provisioning rate (PR) and/or nestling period (NP; Drummond, 2002; González‐Voyer et al., 2007), and indirectly by body mass (BM). The design of path analysis started by defining three basic evolutionary layouts, which differed in the root variable that was hypothesized to act as the primary cause of all other variables: PR (Layout X), BM (Layout Y), or CS (Layout Z) which correspond to the three evolutionary scenarios above (Figure 3 and Appendix S2).

## 3 RESULTS

### 3.1 Broodmate aggression and life history traits

**Figure 4:**
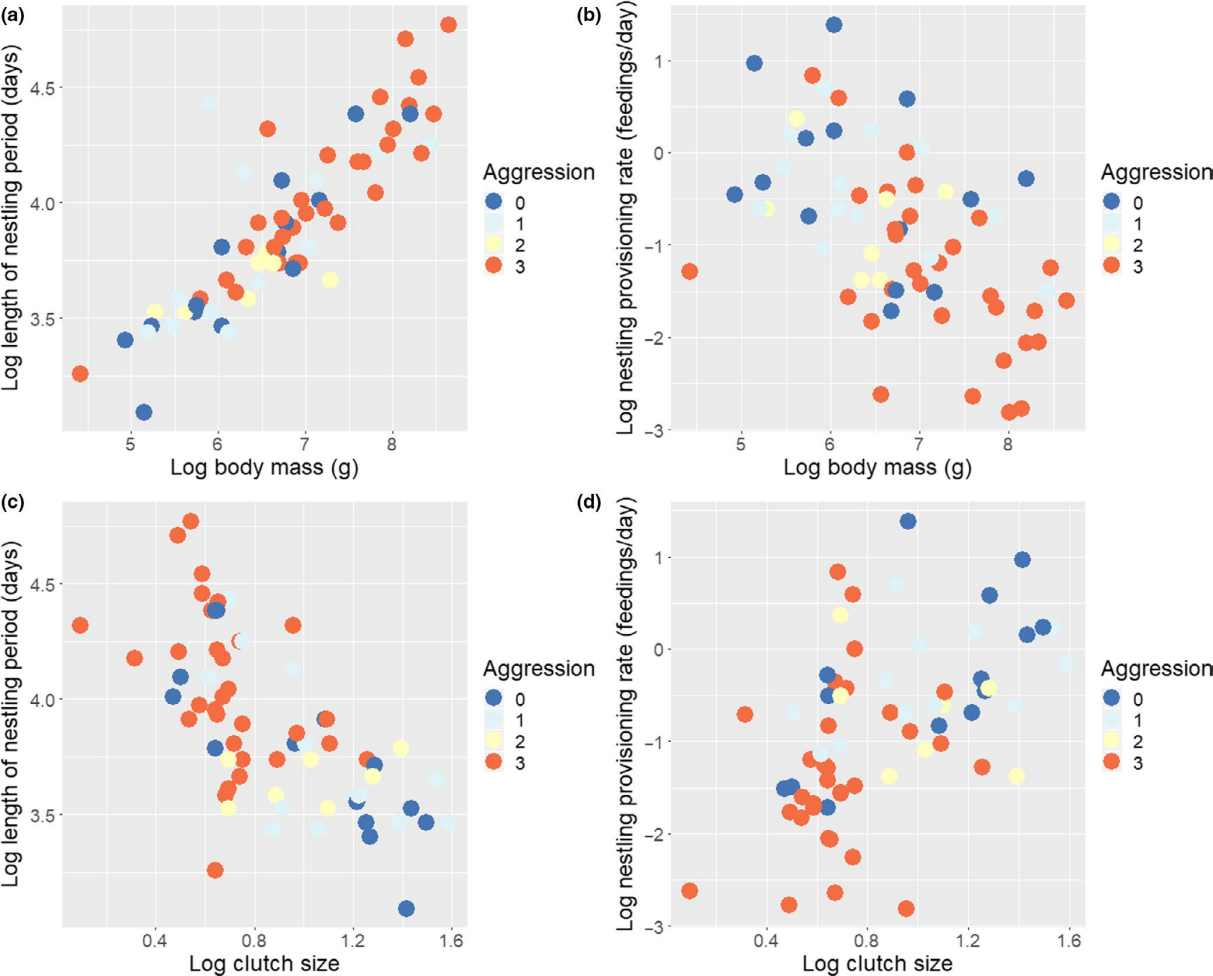
Covariation between the intensity of broodmate aggression (in a colour scale) and life history traits in accipitrid raptors (in logarithmic scale). Upper: Adult body mass in relation to aggression and (a) length of nestling period and (b) nestling provisioning rate. Lower: Clutch size in relation to aggression and (c) length of nestling period and (d) nestling provisioning rate

Aggressive sibling competition was observed in 51 out of 65 species (78%) of accipitrid raptors included in this study. In about half of the species (31/65, 48%), aggressive episodes among broodmates were reported to be frequent and/or extreme (intensity 3), with nonaggressive species (intensity 0) representing a minority (14/65, 21%). Variation in the intensity of broodmate aggression correlated with variation in behavioral and life history traits, which also covaried with each other (Table 1, Figure 4). Aggression was more intense in those species laying smaller clutches and which fed nestlings at lower rates. Moreover, larger species and species with longer nestling periods were more aggressive. These five variables also covaried with each other: Larger species fed nestlings at lower rates, laid smaller clutches, and had longer nestling periods. The intensity of broodmate aggression showed no relationship with feeding method. Feeding method neither showed any significant relationship with the remaining traits (Table 1).

**Table 1:** Relationships among phenotypic traits related to broomate aggression in accipitrid raptors

Model	*λ*	*β*	*SE*	*t*	*df*	*p*	Spearman	*p*
Aggression ~ provisioning rate	.00	−.47	.11	−4.23	63	**<.001**	−.48	**<.001**
Aggression ~ body mass	.00	.36	.11	3.12	63	**.003**	.40	**.001**
Aggression ~ clutch size	.00	−.45	.11	−3.99	63	**<.001**	−.40	**<.001**
Aggression ~ nestling period	.00	.33	.12	2.78	63	**.007**	.36	**.003**
Aggression ~ feeding method	.37	−.17	.13	−1.37	55	.177	−.19	.154
Aggression ~ provisioning rate + body mass	.00	−.38	.13	−2.92	62	**.005**		
Aggression ~ clutch size + body mass	.00	−.35	.13	−2.77	62	**.007**		
Aggression ~ nestling period + body mass	.00	.07	.22	.31	62	.759		
Provisioning rate ~ body mass	.43	−.50	.12	−4.14	63	**<.001**	−.566	**<.001**
Provisioning rate ~ clutch size	.73	.44	.13	3.24	63	**.002**	.562	**<.001**
Provisioning rate ~ nestling period	.43	−.59	.11	−5.10	63	**<.001**	−.618	**<.001**
Provisioning rate ~ feeding method	.88	−.15	.10	−1.48	55	.145	−.115	.393
Provisioning rate ~ clutch size + body mass	.58	.36	.12	2.87	62	**.006**		
Provisioning rate ~ nestling period + body mass	.44	−.49	.18	−2.69	62	**.009**		
Feeding method ~ body mass	.00	−.14	.13	−1.03	55	.308	−.181	.178
Feeding method ~ clutch size	.00	−.15	.13	−1.11	55	.269	−.06	.630
Feeding method ~ nestling period	.00	.06	.12	−.43	55	.665	−.207	.121
Clutch size ~ body mass	.78	−.09	.13	−.75	63	.456	−.533	**<.001**
Clutch size ~ nestling period	.75	−.41	.12	−3.58	63	**<.001**	−.680	**<.001**
Nestling period ~ body mass	.00	.85	.07	12.88	63	**<.001**	.838	**<.001**

Note

Shown are regression models while controlling for phylogeny (PGLS) between behavioural variables (intensity of broodmate aggression, nestling provisioning rate, and feeding method) and their behavioural and life history predictors (sometimes including body mass as a covariate), as well as among life history predictors. Estimates for phylogenetic signal (*λ*), standardized regression coefficients *β* (±*SE*) and their associated *p* values are given. Models with *p* < .01 appear in bold. *N* = 65 species except for models including feeding method (*N* = 57).

After controlling for adult body mass and phylogeny, both the effect of clutch size and nestling provisioning rate on the intensity of broodmate aggression remained significant. However, the length of nestling period failed to have a significant effect on the intensity of aggression when body mass was included in the model. On the contrary, both clutch size and nestling period had an effect on nestling provisioning rate when body mass was controlled for (Table 1).

An IT‐based model selection approach helped to infer the relative importance of these factors as predictors of broodmate aggression. A candidate model set with five predictors comprised 25 models, of which six were truly competitive with ΔAICc < 10 (Table 2). The best model (model 1) showed that intensity of aggression depends on clutch size, provisioning rate, and feeding method. Adding nestling period (model 2) or body mass (model 3) as an extra parameter did not improve the log‐likelihood value, suggesting that these predictors are not informative. The model probability of the first model, after removing models 2 and 3 from the set, changes to 0.63, which represents a 7.8 evidence ratio in favor of the best model relative to the best‐ranked model not including provisioning rate (model 5). Model comparisons revealed that nestling provisioning rate was a powerful predictor of the intensity of broodmate aggression. Provisioning rate was included as a predictor as often as clutch size in the best model subset (4 out of 6 models), followed by feeding method (3 out of 6). The predictive value of feeding method alone (model 25) was poor compared with provisioning rate and clutch size, as revealed by evidence ratios of simple regressions containing each of these predictors relative to a null model with no predictors at all (model 24; models 10, 15, and 25 with evidence ratios 681, 339, and <1, respectively). Feeding method, however, helped explaining some residual variation in the intensity of broodmate aggression, in addition to provisioning rate and clutch size, as suggested by the 4 evidence ratio of a model including feeding method (model 1) relative to a model not including it (model 4). No model containing length of the nestling period or body mass as predictors was selected as part of the best subset. Repeating the analysis with the large dataset of 65 species and four predictors (i.e., feeding method not included) rendered a similar result (Appendix S3). None of the five truly competitive models with ΔAICc < 10 included length of the nestling period as a predictor. Body mass was included in two models but with a very low evidence ratio (12) in favor of models not including it.

**Table 2:** Comparison of multiple regression models of intensity of aggression (response variable) and their life history predictors when controlling for phylogeny using PGLS, ordered by AICc values

Model	Predictors	*K*	AICc	ΔAICc	*L* (g/data)	Weight
1	**Clutch size + provisioning rate + feeding method**	**5**	**145.91**	**0.000**	**1.000**	**0.314**
2	Clutch size + provisioning rate + feeding method + nestling period	6	146.20	0.296	0.862	0.271
3	Clutch size + provisioning rate + feeding method + body mass	6	148.09	2.183	0.336	0.105
4	**Clutch size + provisioning rate**	**4**	**148.77**	**2.866**	**0.239**	**0.075**
5	**Clutch size + feeding method**	**4**	**150.02**	**4.112**	**0.128**	**0.040**
6	**Provisioning rate + feeding method**	**4**	**150.24**	**4.336**	**0.114**	**0.036**
7	Clutch size + provisioning rate + nestling period	5	150.38	4.474	0.107	0.034
8	Clutch size + provisioning rate + body mass	5	151.18	5.272	0.072	0.023
9	**Provisioning rate**	**3**	**151.35**	**5.442**	**0.066**	**0.021**
10	Clutch size + feeding method + body mass	5	152.24	6.335	0.042	0.013
11	Clutch size + feeding method + nestling period	5	152.37	6.459	0.040	0.012
12	Provisioning rate + feeding method + body mass	5	152.55	6.646	0.036	0.011
13	Provisioning rate + feeding method + nestling period	5	152.64	6.736	0.034	0.011
14	**Clutch size**	**3**	**152.75**	**6.838**	**0.033**	**0.010**
15	Provisioning rate + body mass	4	153.23	7.323	0.026	0.008
16	Provisioning rate + nestling period	4	153.58	7.672	0.022	0.007
17	Clutch size + body mass	4	154.18	8.269	0.016	0.005
18	Clutch size + nestling period	4	155.01	9.103	0.011	0.003
19	**Nestling period**	**3**	**160.96**	**15.053**	**0.001**	**0.000**
20	**Body mass**	**3**	**161.01**	**15.105**	**0.001**	**0.000**
21	Nestling period + feeding method	4	161.57	15.666	0.000	0.000
22	Body mass + feeding method	4	162.12	16.212	0.000	0.000
23	Nestling period + body mass	4	162.88	16.970	0.000	0.000
24	**Null**	**2**	**164.40**	**18.490**	**0.000**	**0.000**
25	**Feeding method**	**3**	**164.72**	**18.817**	**0.000**	**0.000**

Note

Truly competitive models appear in bold. *N* = 57 species.

*K*, number of parameters; AICc, Akaike's information criterion with correction for small sample sizes; *L* (g/data), relative likelihood of a model given the data; weight, probability of each model given the data and the set of models being compared.

Provisioning rate was indeed the most important predictor of the intensity of nestmate aggression, as measured by its standardized estimate, followed by clutch size and feeding method (Table 3). In summary, the intensity of broodmate aggression among accipitrid raptors is strongly associated with a low nestling provisioning rate and a small clutch size and, to a lesser extent, a more indirect feeding method. Adult body mass and the duration of nestling period are relatively unimportant factors, and their association with aggression is likely the result of correlations with the two main predictors.

**Table 3:** Model‐averaged parameter estimates *β*, standardized by their partial standard deviations, and their standard errors *SE*, and variable importance for life history predictors of the intensity of broodmate aggression

Predictor	Dataset (number of species)					
	57 spp.			65 spp.		
	*β*	*SE*	Importance	*β*	*SE*	Importance
Provisioning rate	−.280	.117	1.00	−.272	.112	1.00
Clutch size	−.272	.116	0.97	−.226	.105	0.83
Feeding method	−.238	.108	0.85		Not included	
Body mass	Not selected			.014	.015	0.05
Nestling period	Not selected			Not selected		

### 3.2 Phylogenetic path analysis

The candidate set included 1,225 solvable paths, of which 53 fit the observations (nonsignificant *C* statistic), 21 of them being truly competitive models (Table 4). The best‐supported path model (model 1) suggests that a small clutch size is the ultimate direct causal factor responsible for a long nestling period and a low nestling provisioning rate, and indirectly a large body mass (via provisioning rate), which in turn causes a long period of attachment to the nest. Model 1 thus gives support to the *Fecundity* evolutionary scenario. In addition, model 1 suggests that broodmate aggression is directly caused by clutch size and provisioning rate.

**Table 4:** Summary of the Phylogenetic Path Analysis results for the best subset of hypothetical cause‐effect models accounting for the relationship between life history variables and the intensity of broodmate aggression in 65 species of accipitrid raptors

Model	Name^a^	*C*	CICc	*p*	ΔCICc	*L* (g/data)	Weight	Scenario	Direct^b^
1	Yp2s2t2	8.17	38.17	.226	0.000	1.000	0.128	Fecundity	PR, CS
2	Yp2s2t4	8.83	38.83	.183	0.666	0.717	0.092	Fecundity	PR, NP
3	Yp2s2t6	12.21	39.19	.142	1.022	0.600	0.077	Fecundity	PR
4	Xp1s7t2	9.56	39.56	.144	1.393	0.498	0.064	Provisioning	PR, CS
5	Yp2s1t2	9.56	39.56	.144	1.393	0.498	0.064	Allometry	PR, CS
6	Zp2s2t2	9.56	39.56	.144	1.393	0.498	0.064	Fecundity	PR, CS
7	Yp2s6t2	12.94	39.92	.114	1.757	0.415	0.053	Fecundity	PR, CS
8	Xp1s7t4	10.23	40.23	.115	2.059	0.357	0.046	Provisioning	PR, NP
9	Yp2s1t4	10.23	40.23	.115	2.059	0.357	0.046	Allometry	PR, NP
10	Zp2s2t4	10.23	40.23	.115	2.059	0.357	0.046	Fecundity	PR, NP
11	Xp1s7t6	13.60	40.58	.093	2.415	0.299	0.038	Provisioning	PR
12	Yp2s1t6	13.60	40.58	.093	2.415	0.299	0.038	Allometry	PR
13	Zp2s2t6	13.60	40.58	.093	2.415	0.299	0.038	Fecundity	PR
14	Yp2s6t4	13.61	40.59	.093	2.423	0.298	0.038	Fecundity	PR, NP
15	Yp2s6t6	16.98	41.06	.075	2.891	0.236	0.030	Fecundity	PR
16	Xp4s7t2	14.33	41.32	.073	3.149	0.207	0.027	Provisioning	PR, CS
17	Yp2s5t2	14.33	41.32	.073	3.149	0.207	0.027	Allometry	PR, CS
18	Zp2s4t2	14.33	41.32	.073	3.149	0.207	0.027	Fecundity	PR, CS
19	Xp4s7t4	15.00	41.98	.059	3.816	0.148	0.019	Provisioning	PR, NP
20	Yp2s5t4	15.00	41.98	.059	3.816	0.148	0.019	Allometry	PR, NP
21	Zp2s4t4	15.00	41.98	.059	3.816	0.148	0.019	Fecundity	PR, NP

Note

*C*, Fisher's *C* statistics; *k*, number of causal relationships; *p*, *p* value of the *C* statistic; CICc, *C* statistic information criterion corrected for small samples; ΔCICc, difference in CICc from the best fitting model; *L* (g/data), relative likelihood of a model given the data; weight, probability of each model given the data and the set of models being compared (recalculated). Also shown is which of the different evolutionary scenarios is supported by each path.

a Path names define causal relationships according to Appendix S2.

b Life history predictors (BM, body mass; CS, clutch size; PR, provisioning rate) showing a direct causal relationship with the intensity of broodmate aggression.

**Figure 5:**
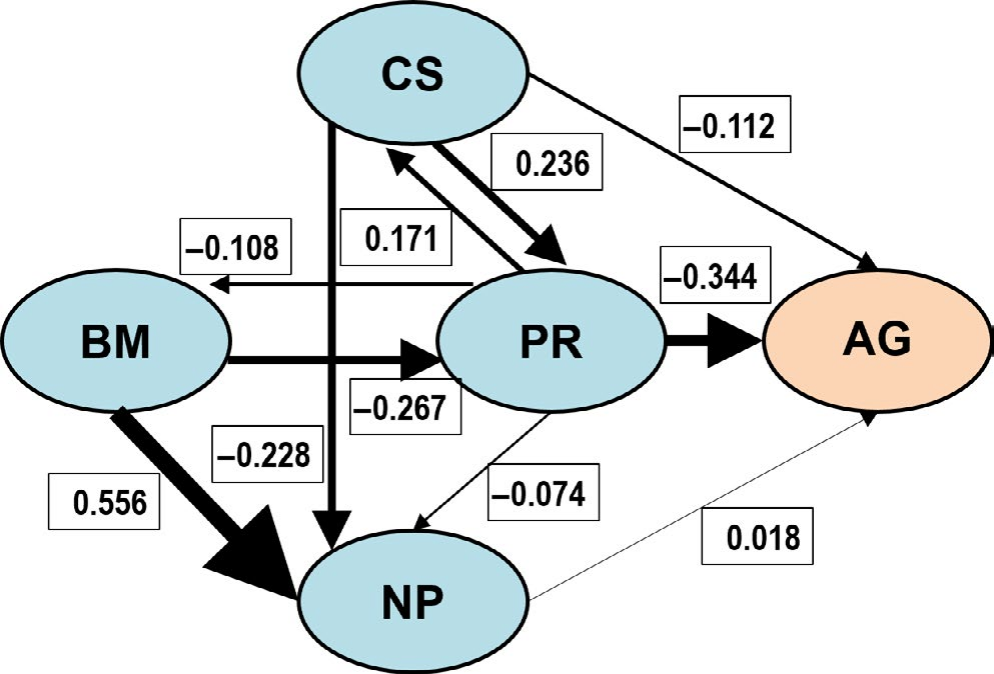
Directed acyclic graph showing the empirical relationships described by the causal model averaged over the subset of models supported by the data (Table 4). The width of the arrows and the numbers represent the value of the standardized regression coefficients, AG, intensity of broodmate aggression; BM, Body Mass (BM); CS, Clutch Size; NP, length of the nestling period; PR, Provisioning Rate

A high degree of uncertainty, however, remains as to which of the plausible models appearing in Table 4 best depicts the causal relationships between nestmate aggression and its life history correlates, thus we computed average parameter and error estimates across all plausible models. These are depicted in Figure 5. Body mass does not show any causal relationship with clutch size but strongly affects nestling period and, to a lesser extent, nestling provisioning rate. Clutch size is the most likely ultimate causal factor giving rise to the observed syndrome of traits related to nestmate aggression. Cumulative evidence in support of the *Fecundity* evolutionary scenario (0.612) is three times that supporting the alternative *Allometry* and *Provisioning* scenarios (0.194) (Table 4). A direct causal effect of provisioning rate on the intensity of broodmate aggression is supported by all models. With respect to clutch size, however, cumulative evidence in support of a direct causal effect on the intensity of broodmate aggression (0.453) is very similar to the amount of evidence indicating an indirect causal effect via provisioning rate, alone or in combination with nestling period (0.470).

### 3.3 Broodmate aggression in relation to foraging lifestyle

Several ecological variables likely to affect prey abundance or availability correlated with life history predictors of broodmate aggression (Table 5). Nestling provisioning rates were lower in species that relied more on warm‐blooded prey, hunted for more agile prey, did not migrate, bred at higher altitudes, and during extended breeding seasons. Low provisioning rates were also associated to less seasonal (low annual range and *SD* of GPP) and more stable habitats (*SD* in all GPP values), particularly those where minimum productivity was also high (average min GPP) and stable (*SD* of min GPP). Smaller clutches were also associated to species breeding in less productive (mean GPP), more stable habitats with extended breeding seasons where production during the least productive month was also high (average min GPP) and less variable (*SD* of min GPP; Table 5). Finally, indirect parental feeding was associated to migratory species breeding in variable habitats with more productive breeding seasons (average max and breeding GPP). Two of these ecological variables had a marginally significant effect upon the intensity of broodmate aggression: Species hunting for more agile prey and breeding in habitats with extended breeding seasons were more aggressive (Table 5).

**Table 5:** The relationship between the intensity of broodmate aggression and its three important behavioral and life history correlates (as response variables) and their ecological predictors

Ecological predictor	Aggression (*λ* < 0.36)			Provisioning rate (*λ* in [0.63, 0.88])			Clutch size (*λ* in [0.80, 0.90])			Feeding method (*λ* = 0.00 for all)		
	*β*	*SE*	*p*	*β*	*SE*	*p*	*β*	*SE*	*p*	*β*	*SE*	*p*
Spatial distribution												
Length of breeding^a^	**.24**	**.12**	**.049**	**−.32**	**.10**	**.003**	**−.24**	**.08**	**.005**	.14	.14	.292
Migration^a^	−.03	.13	.821	**.34**	**.10**	**.001**	**.26**	**.08**	**.003**	**−.31**	**.13**	**.021**
Maximum altitude^a^	.19	.12	.119	**−.29**	**.09**	**.003**	−.11	.09	.221	−.02	.14	.863
Diet												
Agile prey^a^	.22	.13	.090	**−.36**	**.11**	**.002**	.02	.10	.846	−.13	.13	.333
Warm‐blooded prey^a^	−.12	.13	.379	**.34**	**.12**	**.004**	−.06	.11	.601	.02	.13	.901
Diet breadth	−.04	.12	.735	.04	.10	.677	.07	.09	.415	−.12	.13	.366
Habitat productivity												
Average min GPP^a^	.14	.12	.246	**−.24**	**.09**	**.013**	**−.30**	**.08**	**.000**	.22	.13	.099
Average GPP breeding^a^	.11	.12	.379	.11	.12	.364	**.03**	.10	.761	**−.33**	**.12**	**.010**
Average max GPP	.07	.13	.569	.15	.11	.188	.07	.10	.472	**−.31**	**.13**	**.018**
Mean GPP	.17	.12	.162	−.14	.11	.194	**−.24**	**.09**	**.007**	−.11	.13	.400
Habitat variability												
Annual range GPP^a^	−.02	.13	.900	**.26**	**.10**	**.015**	**.25**	**.09**	**.005**	**−.32**	**.13**	**.014**
*SD* GPP (all values)	−.02	.12	.903	**.25**	**.10**	**.016**	**.25**	**.09**	**.005**	**−.32**	**.13**	**.013**
Annual *SD* GPP	−.01	.12	.931	**.25**	**.10**	**.016**	**.25**	**.09**	**.005**	**−.32**	**.13**	**.013**
*SD* min GPP^a^	.13	.12	.281	−.19	.10	.064	**−.29**	**.08**	**.001**	.25	.13	.061
*SD* GPP breeding	.04	.13	.752	.16	.11	.134	**.21**	**.09**	**.022**	−.21	.13	.111
*SD* max GPP	−.05	.12	.707	.04	.10	.722	.11	.08	.206	−.21	.13	.117

Note

Shown are multiple regression models while controlling for phylogeny (PGLS). Estimates for phylogenetic signal (*λ*), standardized regression coefficients *β* (±*SE*), and their associated *p* values are given. Models with *p* < .05 appear in bold. *N* = 65 species except for models including feeding method (*N* = 57).

a Variables selected as predictors in IT‐based model selection procedures.

Hunting success was lower for species relying on more agile prey (PGLS, *β* = −.48 ± .13 *SE*, *t*_38_ = −3.68, *p* < .001, *λ* = 0) and showed a positive relationship with nestling provisioning rates (*β* = .33 ± .14 *SE*, *t*_38_ = 2.45, *p* = .019, *λ* = .81), but it was unrelated to either clutch size (*β* = .02 ± .12 *SE*, *t*_38_ = 0.18, *p* = .860, *λ* = .83) or the intensity of nestling aggression (*β* = −.15 ± .16 *SE*, *t*_38_ = −0.93, *p* = .360, *λ* = .83).

An IT‐based model selection procedure was performed to determine the relative importance of ecological variables as predictors of the intensity of broodmate aggression. To reduce the number of models in the candidate model set, some significant predictors in Table 5 were excluded as redundant. Mean GPP was excluded from the selected predictors because it was highly correlated with average min GPP (*r*_S_ = .71) and average GPP during the breeding season (*r*_S_ = .74). Average max GPP was also removed because it was highly correlated with average GPP during the breeding season (*r*_S_ = .86). Very high correlations were also detected between measures of annual seasonality (annual range and *SD*; *r*_S_ = .99), so we also excluded annual *SD*. Annual range was also strongly correlated with variation during the breeding season (*SD* in GPP breeding, *r*_S_ = .95), so we excluded the latter too. Overall habitat variability in GPP values (*SD* in GPP‐all values) was also excluded because it was highly correlated with all other measures of habitat stability (*r_S_* range .95–.99) except between‐year variation in the least productive month (*SD* in min GPP, *r*_S_ = −.54, *p* < .001). The nine selected ecological variables accounted for variation in spatial distribution of breeding habitats (length of breeding season, maximum altitude, and migration), diet (reliance on agile and warm‐blooded prey), habitat productivity (average min GPP and average GPP during the breeding season), seasonality (annual range of GPP), and between‐year variation in habitat productivity (*SD* in min GPP).

The candidate model set included 2048 models defined by different combinations of the nine selected ecological predictors in Table 5 altogether with the two more important life history predictors (provisioning rate and clutch size). Of these, 512 candidate models did not contain any life history predictors (including a null, intercept‐only model), while the remaining 1536 models contained different combinations of life history and ecological predictors. The candidate model set can be accessed as a dataset (Dryad, https://doi.org/10.5061/dryad.8h07878).

From this candidate model set, we selected a subset of 19 truly competing models (Table 6). In the absence of any information about clutch size and provisioning rate (models 15 to 18), the intensity of nestmate aggression could be predicted by three ecological variables. Species breeding in habitats with extended breeding seasons and hunting more agile prey were more aggressive, as well as (but with higher AICc), species breeding at higher altitudes. Ecological variables, however, were relatively less important when the two life history predictors were included in models (Table 7). Of the three ecological predictors commented above, only reliance on agile prey was of some importance, suggesting that length of breeding season and maximum altitude were actually affecting life history traits. Together with reliance on agile prey, other variables of certain importance in addition to life history traits were seasonality (annual range in GPP), migration and habitat productivity (average GPP during the breeding season). After controlling for life history traits, more aggressive species were migratory species that bred in more seasonal habitats with a highly productive breeding season.

**Table 6:** Comparison of truly‐competitive multiple regression models of intensity of aggression (response variable) and their ecological and life history predictors when controlling for phylogeny using PGLS, ordered by AICc values

Model	Predictors	*K*	AICc	ΔAICc	*L *(g/data)	Weight	
1	Clutch size + provisioning rate + migration	5	169.49	0.000	1.000	0.201	
2	Clutch size + provisioning rate + average GPP breeding	5	169.68	0.187	0.911	0.183	
3	Clutch size + provisioning rate + annual range GPP	5	169.81	0.319	0.853	0.171	
4	Clutch size + provisioning rate	4	170.62	1.133	0.567	0.114	
5	Clutch size + agile prey + average min GPP + annual range GPP	6	171.59	2.094	0.351	0.071	
6	Clutch size + agile prey	4	171.95	2.460	0.292	0.059	
7	Provisioning rate + average GPP breeding	4	172.81	3.319	0.190	0.038	
8	provisioning rate + migration + *SD* min GPP	5	172.97	3.479	0.176	0.035	
9	Provisioning rate + migration	4	173.57	4.077	0.130	0.026	
10	Provisioning rate	3	173.58	4.085	0.130	0.026	
11	Clutch size + warm‐blooded prey + average GPP breeding	5	173.82	4.330	0.115	0.023	
12	Clutch size + warm‐blooded prey + average min GPP + annual range GPP	6	173.87	4.383	0.112	0.022	
13	Clutch size + warm‐blooded prey	4	174.31	4.814	0.090	0.018	
14	Clutch size	3	175.19	5.703	0.058	0.012	
15	Length of breeding + agile prey	4	185.33	15.836	0.000		0.293
16	Length of breeding	3	185.83	16.338	0.000		0.228
17	Agile prey	3	186.17	16.683	0.000		0.192
18	Maximum altitude	3	186.61	17.121	0.000		0.154
19	Null	2	186.93	17.436	0.000		0.132

Note

*K*, number of parameters; AICc, Akaike's statistic information criterion with correction for small sample sizes; *L* (g/data), relative likelihood of a model given the data; weight, probability of each model given the data and the set of models being compared, recalculated after excluding nontruly competitive models (those which are more complex versions of nested models with a lower AICc). Weights for models in analysis either excluding (models 15–19) or including (models 1–14) life history predictors were computed separately.

**Table 7:** Model‐averaged parameter estimates *β*, standardized by their partial standard deviations, and their standard errors *SE*, and variable importance for life history predictors of the intensity of broodmate aggression

Predictor	Predictors included					
	Ecological			Ecological + Life History		
	*β*	*SE*	Importance	*β*	*SE*	Importance
Length of breeding	.126	.088	1.00	Not selected		
Agile prey	.100	.079	0.79	.037	.035	0.13
Maximum altitude	.029	.031	0.23	Not selected		
Clutch size	—			−.277	.123	1.00
Provisioning rate	—			−.273	.110	0.98
Annual range GPP	Not selected			.055	.050	0.20
Migration	Not selected			.052	.048	0.19
Average GPP breeding	Not selected			.047	.044	0.17
Average min GPP	Not selected			.017	.018	0.06
Warm‐blooded prey	Not selected			−.015	.015	0.05
*SD* min GPP	Not selected			Not selected		

## 4 DISCUSSION

### 4.1 Predictors of the intensity of broodmate aggression

A novel finding of this study is that the intensity of broodmate aggression in accipitrid raptors is strongly and negatively correlated with nestling provisioning rate. Provisioning rate, as a proxy of food parcel size, was suggested by Drummond (2002) to explain variations in broodmate aggression at a high (family and above) taxonomic level but a previous study (González‐Voyer et al., 2007) failed to support this prediction. They concluded that the sample on which their analysis was based (69 spp. in seven bird families, including accipitrids, with varying degrees of incidence and intensity of broodmate aggression) might not include species with high feeding rates whose food parcels were small enough to make aggression unprofitable. However, our analysis, based on a similar sample size from a single family, included similar values of maximum feeding rates as González‐Voyer et al. (2007) (4 feedings/hr and 3.8 feedings/hr, respectively) and, notwithstanding, we found a strong effect of nestling provisioning rate upon the intensity of broodmate aggression. One possibility is that differences in foraging and nestling feeding techniques among the bird groups considered in the former study might have obscured variation in provisioning rates related to nestling aggression. Together with a low nestling provisioning rate, and second in importance, intense broodmate aggression was also associated with a small clutch size, followed by an indirect feeding method, which is in agreement with the previous study (González‐Voyer et al., 2007). Feeding method alone was a poor predictor of the intensity of broodmate aggression, but it had some explanatory power when in combination with the two most important predictors. Results relative to feeding method in our study, however, should be taken cautiously, as long as they may rely on biased estimates as a result of heterogeneity in sampling effort. A switch from direct to indirect feeding has been reported to be associated with an increase in broodmate aggression in broad‐winged hawk (*Buteo platypterus*) and Eurasian sparrowhawk (*Accipiter nisus*; Brown & Amadon, 1968; Matray, 1974). In Northern goshawks (*A. gentilis*), aggressive sibling rivalry peaks when attending mothers cease to actively feed the young (Byholm & Kekkonen, 2008; Byholm, Rousi, & Sole, 2011).

Our quantitative analysis confirms earlier descriptive claims that larger species of raptors with longer breeding cycles and laying smaller clutches are more aggressive (Godfray, 1986; Newton, 1977; Simmons, 1988). Intense broodmate aggression is negatively associated with traits (provisioning rate and clutch size) that covary inversely with adult body mass and the duration of postnatal development. However, provisioning rate and clutch size had a much more important role in explaining variations in aggression intensity than adult body mass and the length of the nestling period. The effect of adult body mass upon the intensity of broodmate aggression had never been tested previously. In their comparative study, González‐Voyer et al. (2007) included egg size as a proxy for hatchling mass but egg size is indeed a good proxy for adult mass too (*r*_s_ = .97, *N* = 430, *p* < .001, computed from data in Juang et al., 2017). They found that egg size was not correlated with either the incidence or intensity of aggression while, in contrast with our results, length of the fledging period explained both. We found that the effect of the length of the nestling period on broodmate aggression vanished when we controlled for adult mass, but the latter strongly covaried with the two more important predictors. This suggests the existence of a complex causal structure among the four predictors considered. Correlational analyses of this kind fail, however, to distinguish between alternative causal models (Partridge & Harvey, 1988). Following von Hardenberg and González‐Voyer (2013), we overcame this difficulty by using Phylogenetic Path Analysis to test three alternative evolutionary scenarios. Results of path analysis showed that length of the nestling period not only showed an obvious direct causal relationship with adult mass (*β* = .56) but also with clutch size (*β* = −.23) and that body mass had a strong direct causal link to provisioning rate (*β *= −.27). This, together with a weak causal link between length of the nestling period and the intensity of aggression (*β* = .02), suggests that the association of broodmate aggression with a large body mass and a long fledging period may simply reflect the fact that larger species take longer to fledge and, simultaneously, lay smaller clutches and feed nestlings at lower rates, but only the latter two traits have a direct causal relationship with aggression. Our results do not support a strong direct causal link between the duration of nestling period and the intensity of broodmate aggression for accipitrid raptors (c.f. von Hardenberg & González‐Voyer, 2013).

Path analysis revealed that the observed syndrome of traits was not simply a by‐product of selection primarily operating on body size (the *Allometry* scenario). Body mass showed a direct causal link with provisioning rate and, especially, length of nestling period, but not with clutch size. This result is in accordance with previous comparative studies that found considerable variation in fecundity independently from body mass (Bennett & Owens, 2002; Sibly et al., 2012). It also agrees with the study of von Hardenberg and González‐Voyer (2013), in which (egg) size had only an indirect causal effect upon broodmate aggression. Also in accordance with previous studies (Bennett & Owens, 2002), we found that food abundance (the *Provisioning* scenario) was an unlikely ultimate explanation for covariation among all other traits. Provisioning rate was likely a direct cause of clutch size (*β* = .17), but the opposite was even more likely (*β* = .24), and it was only weakly related to the length of the nestling period (*β* = −.07). Finally, clutch size showed a strong direct causal link both with provisioning rate (*β* = .24) and nestling period (*β* = −.23), suggesting *Fecundity* as the most plausible evolutionary scenario. Cumulative evidence in support of the *Fecundity* scenario, as suggested by IT‐based model selection, was three times that supporting its alternatives. The observed two‐way causal relationship between clutch size and provisioning rate is difficult to explain under the *Provisioning* scenario, but it is expected under the *Fecundity* scenario if both clutch size and provisioning rate are correlated measures of parental effort linked ultimately to a species demography, that is, age‐specific mortality rates (Bennett & Owens, 2002; Martin, 1995, 2004; Ricklefs, 2000). And, finally, only the *Fecundity* scenario predicts a strong, negative direct causal relationship between clutch size and nestling period, in accordance with life history theory, where a positive correlation is expected between adult mortality and growth rates in birds (Martin, 2002; Remeš, 2007). In the study by von Hardenberg and González‐Voyer (2013), a causal link going from egg size to clutch size was hypothesized for all cause‐effect models tested, and provisioning rate was not included as a predictor, hence no comparisons with this study are possible.

### 4.2 Broodmate aggression and cost‐effectiveness

Hypotheses based on cost‐effectiveness assume that costly aggression is only adaptive among those species with a favorable combination of traits at the nestling stage (Mock & Parker, 1997). These hypotheses would predict a strong direct causal link between the intensity of aggression and those predictors making it profitable, e.g. brood size or provisioning rate. In our study, we found a clear negative relationship between the intensity of broodmate aggression and clutch size, mainly because few (5/38, 13%) aggressive species (intensity > 1) laid clutches of 3 eggs or larger, but still a considerable proportion (11/27, 41%) of species with little or no broodmate aggression (intensity < 2) laid clutches of less than 2.5 eggs. However, results from path analysis barely supported a direct causal effect of clutch size on the intensity of broodmate aggression, as compared to an indirect causal effect via provisioning rate, alone or in combination with nestling period. Moreover, the hypothesis that chicks behave more aggressively when accompanied by a smaller number of broodmates has received little experimental support (Drummond & Rodríguez, 2009). This finding seems at odds with a general trend for aggression to be weaker in species with larger broods (Drummond, 2001; González‐Voyer et al., 2007; and this study), although brood (or clutch) size alone may also be of little predictive value for explaining broodmate aggression in other bird taxa, such as herons and storks (Ciconiiformes, Romero & Redondo, 2017). By contrast, a direct causal effect of provisioning rate on aggression was strongly supported by path analysis. It is still unclear, however, whether in the specific case of accipitrid raptors, this result lends support to a higher cost‐effectiveness of aggression when provisioning rates are low, as suggested by the food parcel size hypothesis (Drummond, 2002). The reason is that, among raptors, a large fraction of the food ingested by nestlings is delivered by the female parent, who tears up the available prey into small shreds, which are fed to the chicks in several bouts or meals (Brown & Amadon, 1968). Maternal feeding is most frequent during the first half of the nestling period, coinciding with the onset of broodmate aggression, which usually starts shortly after hatching (Drummond, 2001). The prey items are usually larger than the food required for a single meal, particularly in species capturing large prey, and, while prey size may vary substantially, the relative size of meals is likely relatively consistent among species (Bortolotti, 1986a). Therefore, it is unclear how parcel size received by chicks relates to the variable actually analysed, that is, provisioning rate. Likely, a forthcoming, more rigorous quantitative approach will be needed to resolve how parcel size relates to provisioning rate in accipitrids.

### 4.3 Broodmate aggression as a trait linked to a slow life‐history pace

An alternative explanation to cost‐effectiveness at the nestling stage for the occurrence of covariation between the intensity of broodmate aggression and certain life history traits was advanced by Simmons (1988, 1991), who suggested that intense aggression was an adaptation to maximize juvenile survival and competitive ability in long‐lived species with an intense competition for breeding sites due to habitat saturation. Despite Simmons' (1988) idea is highly cited, and sometimes invoked as an explanation for observed patterns of fertility or broodmate aggression (Bosch, 2003; De Lucca & Saggese, 1995; Watson, Razafindramanana, Thorstrom, & Rafanomezantsoa, 1999), few studies have tested it critically (Simmons, 1993, 2002; Viñuela, 1999), with mixed results. Certainly, it was unfortunate that Simmons's hypothesis was framed in terms of density‐dependent mortality and resource‐limited fecundity at a time when the evolutionary study of life histories was shifting its focus from an *r*/*K*‐selection to a demographic theory paradigm (Partridge & Harvey, 1988; Reznick, Bryant, & Bashey, 2002; Wilbur, Tinkle, & Collins, 1974). Moreover, the hypothesis was later dismissed because it was considered an example of “progeny choice” explanation for the evolution of broodmate aggression, which is unlikely to work in the case of asynchronously‐hatching birds (Forbes & Mock, 1998; Mock, 2004), and because studies in other bird groups failed to support some of its assumptions, For example that fratricide provides senior chicks with extra food (Ploger, 1997) and that variations in clutch size are adapted to track changes in population density (Nelson, 1989).

Notwithstanding these criticisms, our results fit remarkably well with Simmons' (1988, 1991) original claim that intense broodmate aggression in accipitrid raptors is a behavioral trait characteristically associated to a slow life history pace (Sæther et al., 2013), that is, a low reproductive effort (low fertility and restrained parental expenditure) suggesting a long life span and a high subadult‐to‐adult mortality ratio. Among birds, longevity and adult survival are negatively correlated with annual fertility (eggs/year; Bennett & Harvey, 1988; Bennett & Owens, 2002; Ricklefs, 2000) or clutch size (Linden & Møller, 1989; Martin, 1995) and with parental effort in nest attentiveness (Martin, 2002), chick provisioning (Bortolotti, 1986a) and anti‐predator defence (Ghalambor & Martin, 2001). Such a relationship is consistent with the idea that where extrinsic factors cause little adult mortality, parental effort is restrained to reduce reproduction‐related mortality and preserve the potentially long life span of individuals (Charlesworth, 1994; Ricklefs, 2000). More specifically, demographic models of life history evolution predict that reproductive effort should decrease if the ratio of extrinsic adult to juvenile (prebreeding) mortality is high (Charnov & Schaffer, 1973; Murphy, 1968; Ricklefs, 2010; Sæther et al., 2013; Stearns, 1976).

As we have shown above, path analysis supported an evolutionary scenario where the intensity of broodmate aggression and its correlated traits among accipitrid raptors were ultimately caused by a slow fecundity, rather than allometric constraints or food limitation, compatible with a low adult mortality. An indirect causal effect of clutch size on aggression is also compatible with this scenario. In the absence of information about parental effort (clutch size and provisioning rate), the intensity of broodmate aggression correlated with three ecological factors. More aggressive species of raptors bred in habitats with extended breeding seasons, at higher altitudes and hunted for more agile prey. Extended breeding seasons are typical of tropical latitudes and less seasonal habitats, and there is a well‐established relationship between avian life history and latitude (Cody, 1966; Martin et al., 2015) or seasonality (Jetz, Sekercioglu, & Böhning‐Gaese, 2008; McNamara, Barta, Wikelski, & Houston, 2008). This relationship was evident in our data sample (Tables 5, 6, 7): A low parental effort was associated with less seasonal, more stable habitats with high and stable primary production during the least productive month. Simmons (1988, 1991) already pointed to a tropical origin as one of the factors characterizing siblicidal raptors. In accordance with this claim, it has been shown that tropical populations of ospreys *Pandion haliaetus* (Poole, 1982) and swallow‐tailed kites *Elanoides forficatus* (Gerhardt, Gerhardt, & Vasquez, 1997) display more intense broodmate aggression than temperate populations. In the case of ospreys, these differences cannot be attributed to a lower food provisioning at lower latitudes (Prevost, 1982). A slower life history is also reported for bird species or populations breeding at high elevations, which tend to produce fewer offspring but increase investment per offspring as a strategy to increase offspring survival (Badyaev & Ghalambor, 2001; Bears, Martin, & White, 2009; Hille & Cooper, 2015). Neither length of the breeding season nor maximum altitude explained variations in the intensity of broodmate aggression when life history predictors were included in the models, again suggesting that effects of the location of breeding habitat were indeed affecting reproductive effort, and not broodmate aggression directly. Finally, more aggressive species also hunted for more agile prey and this effect was still of some importance after variables of parental effort were included in models. Hunting success (and provisioning rates) was lower in species hunting for more agile prey, but it was unrelated to the intensity of broodmate aggression. This again suggests that nestling aggression in these species was not a consequence of food limitation because of adults being inefficient hunters. Moreover, lower provisioning rates in these species may be actually compensated by the fact that more agile prey are typically of a larger relative size (von Schantz & Nilsson, 1981; Toland, 1986). An alternative explanation is that raptor species relying on more agile prey require considerable time and practice to improve foraging skills during their juvenile life (Edwards, 1989; Nadjafzadeh, Hofer, & Krone, 2015; Rutz, 2012; Rutz, Whittingham, & Newton, 2006), compared with species hunting for less agile prey (e.g., American kestrels *Falco sparverius*, Varland, Klass, & Loughin, 1991). This may have a profound impact on life history parameters, particularly by reducing the prebreeding/adult survival ratio (Ashmole, 1963; Wiens, Noon, & Reynolds, 2006) and increasing age at first breeding (Krüger, 2005, 2007), that is, slowing down a species' life history pace. An increase in subadult/adult survival ratio may also explain why, after taking parental effort predictors into account, some other ecological variables were of certain importance to explain variations in the intensity of broodmate aggression. For a similar reproductive effort, more aggressive raptors were migratory species that bred in highly seasonal habitats. Both migration (Rotics et al., 2016; Sergio et al., 2014) and habitat seasonality (Ricklefs, 2000; Tarwater, Ricklefs, Maddox, & Brawn, 2011) are extrinsic causes of mortality that affect juvenile birds disproportionately.

### 4.4 How a slow life‐history pace may select for aggressive broodmate competition

Nestling aggression likely serves to secure a greater share of parental investment by intimidating, dominating, or eliminating competitors (Drummond, 2001; Mock et al., 1990). Aggression may either provide senior chicks with extra food in the short term (Simmons, 2002) or optimize their growth by buffering it against eventual fluctuations in resource availability (Mock, 2004) in order to maximize its chances of postfledging survival (Stinson, 1979). Suboptimal growth negatively affects juvenile survival after leaving the nest (Maness & Anderson, 2013; Naef‐Daenzer & Grüebler, 2016; Remeš & Matysioková, 2016; Schwagmeyer & Mock, 2008), especially after nestlings attain nutritional independence (Dybala, Gardali, & Eadie, 2013; Wiens et al., 2006). Heavier offspring may be able to acquire more food, survive periods of food shortage, or spend less time foraging and more time watching for predators (Briga, Koetsier, Boonekamp, Jimeno, & Verhulst, 2017; Sullivan, 1989). Selection for aggressive competition may be particularly strong in species with a slow life history because (1) restrained parental provisioning effort may not fully compensate for environmental fluctuations in resources (Bókony et al., 2009; Bortolotti, 1986a), and (2) a suboptimal growth has a comparatively higher fitness penalty because lifetime reproductive success depends more on survival than on fertility (Murphy, 1968; Sæther et al., 2013). Thus, a slow life pace history underlies a trait syndrome already recognized since long ago. Intense broodmate aggression is associated with a large body size (allowing a long life span, Healy et al., 2014), restrained parental effort (as a result of a low adult mortality, Sibly et al., 2012), and a complex foraging lifestyle (Sibly & Brown, 2007) characterized by a protracted period of learning during early life, For example, hunting for agile prey, or aerial bone‐dropping by bearded vultures (*Gypaetus barbatus*). In addition, parents in these long‐lived species may be more strongly selected to facilitate the arena for sibling rivalry by promoting competitive asymmetries among offspring (Forbes & Mock, 2000; Mock & Forbes, 1994). This may increase the cost‐effectiveness of aggression in the short term. However, as this study suggests, cost‐effectiveness alone seems a less satisfactory explanation than life‐history pace for interspecific variation in the intensity of broodmate aggression among accipitrid raptors, and possibly other birds.

## CONFLICT OF INTERESTS

The authors declare no conflict of interests.

## AUTHOR CONTRIBUTIONS

T.R., J.M.R., and J.N. designed the study. T.R., J.M.R., and R.D. collected the data. J.N. analysed the data. T.R., J.N., R.D. and J.M.R. wrote the manuscript.

## Data Availability

Data are deposited on Dryad. https://doi.org/10.5061/dryad.8h07878.

## References

[ece35466-bib-0001] Anderson, D. R. (2008). Model based inference in the life sciences: A primer on evidence. New York, NY: Springer.

[ece35466-bib-0002] Arnold, T. W. (2010). Uninformative parameters and model selection using Akaike's Information Criterion. Journal of Wildlife Management, 74, 1175–1178. 10.1111/j.1937-2817.2010.tb01236.x

[ece35466-bib-0003] Ashmole, N. P. (1963). The regulation of numbers of tropical oceanic birds. Ibis, 103, 458–473. 10.1111/j.1474-919X.1963.tb06766.x

[ece35466-bib-0004] Badyaev, A. V. , & Ghalambor, C. K. (2001). Evolution of life histories along elevational gradients: Trade‐off between parental care and fecundity. Ecology, 82, 2948–2960. 10.1890/0012-9658(2001)082[2948:EOLHAE]2.0.CO;2

[ece35466-bib-0005] Balfour, E. (1957). Observations on the breeding biology of the hen harrier in Orkney. Bird Notes, 27, 177–183.

[ece35466-bib-0006] Bears, H. , Martin, K. , & White, G. C. (2009). Breeding in high‐elevation habitat results in shift to slower life‐history strategy within a single species. Journal of Animal Ecology, 78, 365–375. 10.1111/j.1365-2656.2008.01491.x 19007385

[ece35466-bib-0007] Bellino, A. , Baldantoni, D. , De Nicola, F. , Iovieno, P. , Zaccardelli, M. , & Alfani, A. (2015). Compost amendments in agricultural ecosystems: Confirmatory path analysis to clarify the effects on soil chemical and biological properties. The Journal of Agricultural Science, 153, 282–295. 10.1017/S0021859614000033

[ece35466-bib-0008] Bennett, P. M. , & Harvey, P. H. (1988). How fecundity balances mortality in birds. Nature, 333, 216 10.1038/333216b0

[ece35466-bib-0009] Bennett, P. M. , & Owens, I. P. F. (2002). Evolutionary ecology of birds: Life histories, mating systems and extinction. Oxford, UK: Oxford University Press.

[ece35466-bib-0010] BirdLife International (2015). IUCN Red List for Birds. Retrieved from http://www.birdlife.org

[ece35466-bib-0011] Bókony, V. , Lendvai, Á. Z. , Liker, A. , Angelier, F. , Wingfield, J. C. , & Chastel, O. (2009). Stress response and the value of reproduction: Are birds prudent parents? The American Naturalist, 173, 589–598. 10.1086/597610 19281425

[ece35466-bib-0012] Bortolotti, G. R. (1986a). Evolution of growth rates in eagles: Sibling competition vs. energy considerations. Ecology, 67, 182–194. 10.2307/1938517

[ece35466-bib-0013] Bortolotti, G. R. (1986b). Influence of sibling competition on nestling sex ratios of sexually dimorphic birds. The American Naturalist, 127, 495–507. 10.1086/284498

[ece35466-bib-0014] Bosch, J. (2003). Fenología y parámetros reproductivos del aguililla calzada Hieraaetus pennatus en Catalu‐a Central (Espa‐a). Ardeola, 50, 181–189.

[ece35466-bib-0015] Briga, M. , Koetsier, E. , Boonekamp, J. J. , Jimeno, B. , & Verhulst, S. (2017). Food availability affects adult survival trajectories depending on early developmental conditions. Proceedings of the Royal Society of London B: Biological Sciences, 284, 20162287 10.1098/rspb.2016.2287 PMC5247499 28053061

[ece35466-bib-0016] Brown, C. J. (1990). Breeding biology of the bearded vulture in southern Africa, Part I: The prelaying and incubation periods. Ostrich, 61, 24–32. 10.1080/00306525.1990.9633934

[ece35466-bib-0017] Brown, N. , & Amadon, D. (1968). Eagles, hawks and falcons of the world. Vols. 1, 2. New York, NY: McGraw‐Hill.

[ece35466-bib-0018] Burnham, K. P. , & Anderson, D. R. (2002). Model selection and multimodel inference: A practical information‐theoretic approach. New York, NY: Springer.

[ece35466-bib-0019] Byholm, P. , & Kekkonen, M. (2008). Food regulates reproduction differently in different habitats: Experimental evidence in the goshawk. Ecology, 89, 1696–1702. 10.1890/07-0675.1 18589533

[ece35466-bib-0020] Byholm, P. , Rousi, H. , & Sole, I. (2011). Parental care in nesting hawks: Breeding experience and food availability influence the outcome. Behavioral Ecology, 22, 609–615. 10.1093/beheco/arr019

[ece35466-bib-0021] Cade, B. S. (2015). Model averaging and muddled multimodel inferences. Ecology, 96, 2370–2382. 10.1890/14-1639.1 26594695

[ece35466-bib-0022] Calder, W. A. (1984). Size, function, and life history. Cambridge, MA: Harvard University Press.

[ece35466-bib-0023] Charlesworth, B. (1994). Evolution in age‐structured populations. Cambridge, UK: Cambridge University Press.

[ece35466-bib-0024] Charnov, E. L. , & Schaffer, W. M. (1973). Life‐history consequences of natural selection: Cole's result revisited. The American Naturalist, 107, 791–793. 10.1086/282877

[ece35466-bib-0025] Cody, M. L. (1966). A general theory of clutch size. Evolution, 20, 174–184. 10.1111/j.1558-5646.1966.tb03353.x 28563630

[ece35466-bib-0026] De Lucca, E. R. , & Saggese, M. D. (1995). Fratricidio en el águila mora Geranoaetus melanoleucus. El Hornero, 14, 38–39.

[ece35466-bib-0027] Drent, R. H. , & Daan, S. (1980). The prudent parent: Energetic adjustments in avian breeding. Ardea, 68, 225–252.

[ece35466-bib-0028] Drummond, H. (2001). The control and function of agonism in avian broodmates. Advances in the Study of Behavior, 30, 261–301. 10.1016/S0065-3454(01)80009-7

[ece35466-bib-0029] Drummond, H. (2002). Begging versus aggression in avian broodmate competition In Wright J., & Leonard M. L. (Eds.), The evolution of nestling begging: Competition, cooperation and communication (pp. 337–360). Dordrecht, The Netherlands: Kluwer Academic Press.

[ece35466-bib-0030] Drummond, H. (2006). Dominance in vertebrate broods and litters. The Quarterly Review of Biology, 81, 3–32. 10.1086/503922 16602272

[ece35466-bib-0031] Drummond, H. , & Rodríguez, C. (2009). No reduction in aggression after loss of a broodmate: A test of the brood size hypothesis. Behavioral Ecology and Sociobiology, 63, 321–327. 10.1007/s00265-008-0664-7

[ece35466-bib-0032] Dybala, K. E. , Gardali, T. , & Eadie, J. M. (2013). Dependent vs. independent juvenile survival: Contrasting drivers of variation and the buffering effect of parental care. Ecology, 94, 1584–1593. 10.1890/12-1443.1 23951718

[ece35466-bib-0033] Edwards, T. C. (1989). The ontogeny of diet selection in fledgling ospreys. Ecology, 70, 881–896. 10.2307/1941356

[ece35466-bib-0034] Ferguson‐Lees, J. , & Christie, D. A. (2001). Raptors of the world. Boston, MA: Houghton Mifflin Harcourt.

[ece35466-bib-0035] Forbes, L. S. , & Mock, D. W. (1998). Parental optimism and progeny choice: When is screening for offspring quality affordable. Journal of Theoretical Biology, 192, 3–14. 10.1006/jtbi.1997.0596 9628835

[ece35466-bib-0036] Forbes, L. S. , & Mock, D. W. (2000). A tale of two strategies: Life‐history aspects of family strife. The Condor, 102, 23–34. 10.1650/0010-5422(2000)102[0023:ATOTSL]2.0.CO;2

[ece35466-bib-0037] Fox, J. , & Weisberg, S. (2011). An R companion to applied regression, 2nd ed. Thousand Oaks, CA: Sage Retrieved from http://socserv.socsci.mcmaster.ca/jfox/Books/Companion

[ece35466-bib-0038] Freckleton, R. P. (2011). Dealing with collinearity in behavioural and ecological data: Model averaging and the problems of measurement error. Behavioral Ecology and Sociobiology, 65, 91–101. 10.1007/s00265-010-1045-6

[ece35466-bib-0039] Garamszegi, L. Z. (2014). Uncertainties due to within‐species variation in comparative studies: Measurement errors and statistical weights In Garamszegi L. Z. (Ed.), Modern phylogenetic comparative methods and their application in evolutionary biology (pp. 157–199). Berlin, Germany: Springer.

[ece35466-bib-0040] Garamszegi, L. Z. , & Møller, A. P. (2010). Effects of sample size and intraspecific variation in phylogenetic comparative studies: A meta‐analytic review. Biological Reviews, 85, 797–805. 10.1111/j.1469-185X.2010.00126.x 20148861

[ece35466-bib-0041] Garamszegi, L. Z. , & Møller, A. P. (2012). Untested assumptions about within‐species sample size and missing data in interspecific studies. Behavioral Ecology and Sociobiology, 66, 1363–1373. 10.1007/s00265-012-1370-z

[ece35466-bib-0042] Garamszegi, L. Z. , & Mundry, R. (2014). Multimodel‐inference in comparative analyses In Garamszegi L. Z. (Ed.), Modern phylogenetic comparative methods and their application in evolutionary biology (pp. 305–331). Berlin, Germany: Springer.

[ece35466-bib-0043] Gerhardt, R. P. , Gerhardt, D. M. , & Vasquez, M. A. (1997). Siblicide in swallow‐tailed kites. Wilson Bulletin, 109, 112–120.

[ece35466-bib-0044] Ghalambor, C. K. , & Martin, T. E. (2001). Fecundity‐survival trade‐offs and parental risk‐taking in birds. Science, 29, 494–497. 10.1126/science.1059379 11313493

[ece35466-bib-0045] Godfray, H. C. J. (1986). Brood reduction and desertion in herons and egrets. Trends in Ecology and Evolution, 1, 33 10.1016/0169-5347(86)90068-6

[ece35466-bib-0046] Godfray, H. C. J. , & Harper, A. B. (1990). The evolution of brood reduction by siblicide in birds. Journal of Theoretical Biology, 145, 163–175. 10.1016/S0022-5193(05)80122-5

[ece35466-bib-0047] González‐Voyer, A. , & Drummond, H. (2007). Is broodmate aggression really associated with direct feeding? Behaviour, 144, 373–392. 10.1163/156853907780756049

[ece35466-bib-0048] González‐Voyer, A. , Székely, T. , & Drummond, H. (2007). Why do some siblings attack each other? Comparative analysis of aggression in avian broods. Evolution, 61, 1946–1955. 10.1111/j.1558-5646.2007.00152.x 17683436

[ece35466-bib-0049] González‐Voyer, A. , & von Hardenberg, A. (2014). An introduction to phylogenetic path analysis In Garamszegi L. Z. (Ed.), Modern phylogenetic comparative methods and their application in evolutionary biology (pp. 201–229). Berlin, Germany: Springer.

[ece35466-bib-0050] Grueber, C. E. , Nakagawa, S. , Laws, R. J. , & Jamieson, I. G. (2011). Multimodel inference in ecology and evolution: Challenges and solutions. Journal of Evolutionary Biology, 24, 699–711. 10.1111/j.1420-9101.2010.02210.x 21272107

[ece35466-bib-0051] Harrison, X. A. , Donaldson, L. , Correa‐Cano, M. E. , Evans, J. , Fisher, D. N. , Goodwin, C. E. D. , … Inger, R. (2018). A brief introduction to mixed effects modelling and multi‐model inference in ecology. PeerJ, 6, e4794 10.7717/peerj.4794 29844961 PMC5970551

[ece35466-bib-0052] Harvey, P. H. , & Pagel, M. (1991). The evolutionary method in comparative biology. Oxford, UK: Oxford University Press.

[ece35466-bib-0053] Healy, K. , Guillerme, T. , Finlay, S. , Kane, A. , Kelly, S. B. A. , McClean, D. , … Cooper, N. (2014). Ecology and mode‐of‐life explain lifespan variation in birds and mammals. Proceedings of the Royal Society of London B: Biological Sciences, 281, 20140298 10.1098/rspb.2014.0298 PMC4043093 24741018

[ece35466-bib-0054] Hille, S. M. , & Cooper, C. B. (2015). Elevational trends in life histories: Revising the pace‐of‐life framework. Biological Reviews, 90, 204–213. 10.1111/brv.12106 24673806

[ece35466-bib-0055] Huertas, A. , Peri, P. L. , Diaz‐Delgado, R. , & Martínez Pastur, G. (2016). Changes in net primary productivity in Argentine forest regions and according to their conservation status In First IUFRO Landscape Ecology Latin‐American Congress and Second IALE Latin‐American Congress: Book of abstracts (pp. 180). Temuco, Chile: Universidad de la Frontera.

[ece35466-bib-0056] Jetz, W. , Sekercioglu, C. H. , & Böhning‐Gaese, K. (2008). The worldwide variation in avian clutch size across species and space. PLoS Biology, 6, e303 10.1371/journal.pbio.0060303 PMC2596859 19071959

[ece35466-bib-0057] Jetz, W. , Thomas, G. H. , Joy, J. B. , Hartmann, K. , & Mooers, A. O. (2012). The global diversity of birds in space and time. Nature, 491, 444–448. 10.1038/nature11631 23123857

[ece35466-bib-0058] Juang, J. Y. , Chen, P. Y. , Yang, D. C. , Wu, S. P. , Yen, A. , & Hsieh, H. I. (2017). The avian egg exhibits general allometric invariances in mechanical design. Scientific Reports, 7, 14205 10.1038/s41598-017-14552-0 29079743 PMC5660176

[ece35466-bib-0059] Kamilar, J. M. , & Cooper, N. (2013). Phylogenetic signal in primate behaviour, ecology and life history. Philosophical Transactions of the Royal Society B: Biological Sciences, 368, 20120341 10.1098/rstb.2012.0341 PMC3638444 23569289

[ece35466-bib-0060] Komsta, L. , & Novomestky, F. (2015). moments: Moments, cumulants, skewness, kurtosis and related tests. R package version 0.14. Retrieved from https://CRAN.R-project.org/package=moments

[ece35466-bib-0061] Krüger, O. (2005). Age at first breeding and fitness in goshawk Accipiter gentilis. Journal of Animal Ecology, 74, 266–273. 10.1111/j.1365-2656.2005.00920.x

[ece35466-bib-0062] Krüger, O. (2007). Long‐term demographic analysis in goshawk Accipiter gentilis: The role of density dependence and stochasticity. Oecologia, 152, 459–471. 10.1007/s00442-007-0677-3 17356810

[ece35466-bib-0063] Lack, D. (1968). Ecological adaptations for breeding in birds. London, UK: Methuen.

[ece35466-bib-0064] Lamey, T. C. , & Mock, D. W. (1991). Non‐aggressive brood reduction in birds. Acta XX Congressus Internationalis Ornithologici, 3, 1741–1751.

[ece35466-bib-0065] Linden, M. , & Møller, A. P. (1989). Cost of reproduction and covariation of life history traits in birds. Trends in Ecology and Evolution, 4, 367–371. 10.1016/0169-5347(89)90101-8 21227380

[ece35466-bib-0066] Machmer, M. (1992). Causes and consequences of sibling aggression in nestling ospreys Pandion haliaetus. Doctoral dissertation, Thesis. Department of Biological Sciences, Simon Fraser University.

[ece35466-bib-0067] Maness, T. J. , & Anderson, D. J. (2013). Predictors of juvenile survival in birds. Ornithological Monographs, 78, 1–55. 10.1525/om.2013.78.1.1

[ece35466-bib-0068] Martin, T. E. (1995). Avian life history evolution in relation to nest sites, nest predation, and food. Ecological Monographs, 65, 101–127. 10.2307/2937160

[ece35466-bib-0069] Martin, T. E. (2002). A new view of avian life‐history evolution tested on an incubation paradox. Proceedings of the Royal Society of London B: Biological Sciences, 269, 309–316. 10.1098/rspb.2001.1879 PMC1690888 11839200

[ece35466-bib-0070] Martin, T. E. (2004). Avian life‐history evolution has an eminent past: Does it have a bright future? The Auk, 121, 289–301. 10.1642/0004-8038(2004)121[0289:ALEHAE]2.0.CO;2

[ece35466-bib-0071] Martin, T. E. , Martin, P. R. , Olson, C. R. , Heidinger, B. J. , & Fontaine, J. J. (2000). Parental care and clutch sizes in North and South American birds. Science, 287, 1482–1485. 10.1126/science.287.5457.1482 10688796

[ece35466-bib-0072] Martin, T. E. , Oteyza, J. C. , Mitchell, A. E. , Potticary, A. L. , & Lloyd, P. (2015). Postnatal growth rates covary weakly with embryonic development rates and do not explain adult mortality probability among songbirds on four continents. The American Naturalist, 185, 380–389. 10.1086/679612 25674692

[ece35466-bib-0073] Martínez Pastur, G. , Huertas, A. , Rosas, Y. M. , Barrera, M. D. , Amoroso, M. M. , Alcobé, F. , & Peri, P. L. (2018). Influencia del cambio climático en los bosques nativos de Argentina: ¿Qué estrategias de manejo y conservación deberían considerarse? In Libro de Silvicultura del Bosque Nativo en la Argentina, Dirección de Bosques, Ministerio de Ambiente y Desarrollo Sustentable de la Nación. Buenos Aires, Argentina.

[ece35466-bib-0074] Martins, E. P. , & Hansen, T. F. (1997). Phylogenies and the comparative method: A general approach to incorporating phylogenetic information into the analysis of interspecific data. The American Naturalist, 149, 646–667. 10.1086/286013

[ece35466-bib-0075] Matray, P. F. (1974). Broad‐winged hawk nesting and ecology. The Auk, 91, 307–324.

[ece35466-bib-0076] Mazerolle, M. J. (2017). AICcmodavg: Model selection and multimodel inference based on (Q)AIC(c). R package version 2.1‐1. Retrieved from https://cran.r-project.org/package=AICcmodavg

[ece35466-bib-0077] McNamara, J. M. , Barta, Z. , Wikelski, M. , & Houston, A. I. (2008). A theoretical investigation of the effect of latitude on avian life histories. The American Naturalist, 172, 331–345. 10.1086/589886 18666854

[ece35466-bib-0078] Metcalfe, N. B. , & Monaghan, P. (2003). Growth versus lifespan: Perspectives from evolutionary ecology. Experimental Gerontology, 38, 935–940. 10.1016/S0531-5565(03)00159-1 12954479

[ece35466-bib-0079] Mock, D. W. (1985). Siblicidal brood reduction: The prey‐size hypothesis. The American Naturalist, 125, 327–343. 10.1086/284346

[ece35466-bib-0080] Mock, D. W. (2004). More than kin and less than kind: The evolution of family conflict. Cambridge, MA: Belknap Press.

[ece35466-bib-0081] Mock, D. W. , Drummond, H. , & Stinson, C. H. (1990). Avian siblicide. The American Scientist, 78, 438–449.

[ece35466-bib-0082] Mock, D. W. , & Forbes, L. S. (1994). Life‐history consequences of avian brood reduction. The Auk, 111, 115–123. 10.2307/4088510

[ece35466-bib-0083] Mock, D. W. , & Parker, G. A. (1997). The evolution of sibling rivalry. Oxford, UK: Oxford University Press.

[ece35466-bib-0084] Monteith, J. L. (1972). Solar radiation and productivity in tropical ecosystems. Journal of Applied Ecology, 9, 747–766. 10.2307/2401901

[ece35466-bib-0085] Monteith, J. L. (1977). Climate and efficiency of crop production in Britain. Philosophical Transactions of the Royal Society B: Biological Sciences, 281, 277–294. 10.1098/rstb.1977.0140

[ece35466-bib-0086] Murphy, G. I. (1968). Pattern in life history and the environment. The American Naturalist, 102, 391–403. 10.1086/282553

[ece35466-bib-0087] Nadjafzadeh, M. , Hofer, H. , & Krone, O. (2015). Sit‐and‐wait for large prey: Foraging strategy and prey choice of white‐tailed eagles. Journal of Ornithology, 157, 165–178. 10.1007/s10336-015-1264-8

[ece35466-bib-0088] Naef‐Daenzer, B. , & Grüebler, M. U. (2016). Post‐fledging survival of altricial birds: Ecological determinants and adaptation. Journal of Field Ornithology, 87, 227–250. 10.1111/jofo.12157

[ece35466-bib-0089] Nagy, J. , & Tökölyi, J. (2014). Phylogeny, historical biogeography and the evolution of migration in accipitrid birds of prey (Aves: Accipitriformes). Ornis Hungarica, 22, 15–35. 10.2478/orhu-2014-0008

[ece35466-bib-0090] Nagy, J. , Végvári, Z. S. , & Varga, Z. (2017). Life history traits, bioclimate, and migratory systems of accipitrid birds of prey (Aves: Accipitriformes). Biological Journal of the Linnean Society, 121, 63–71. 10.1093/biolinnean/blw021

[ece35466-bib-0091] Nelson, B. (1989). Cainism in the Sulidae. Ibis, 131, 609 10.1111/j.1474-919X.1989.tb04796.x

[ece35466-bib-0092] Newton, I. (1977). Breeding strategies in birds of prey. Living Bird, 16, 51–82.

[ece35466-bib-0093] Newton, I. (1979). Population ecology of raptors. Berkhamsted, UK: T. and A. D. Poyser.

[ece35466-bib-0094] Oatley, G. , Simmons, R. E. , & Fuchs, J. (2015). A molecular phylogeny of the harriers (Circus, Accipitridae) indicate the role of long distance dispersal and migration in diversification. Molecular Phylogenetics and Evolution, 85, 150–160. 10.1016/j.ympev.2015.01.013 25701771

[ece35466-bib-0095] O'Connor, R. J. (1978). Brood reduction in birds: Selection for fratricide, infanticide and suicide? Animal Behaviour, 26, 79–96. 10.1016/0003-3472(78)90008-8

[ece35466-bib-0096] Pagel, M. (1997). Inferring evolutionary processes from phylogenies. Zoological Scripta, 26, 331–348. 10.1111/j.1463-6409.1997.tb00423.x

[ece35466-bib-0097] Pagel, M. (1999). Inferring the historical patterns of biological evolution. Nature, 401, 877–884. 10.1038/44766 10553904

[ece35466-bib-0098] Paradis, E. (2014). An introduction to the phylogenetic comparative method In Garamszegi L. Z. (Ed.), Modern phylogenetic comparative methods and their application in evolutionary biology (pp. 3–18). Berlin, Germany: Springer.

[ece35466-bib-0099] Paradis, E. , Claude, J. , & Strimmer, K. (2004). APE: Analyses of phylogenetics and evolution in R language. Bioinformatics, 20, 289–290. 10.1093/bioinformatics/btg412 14734327

[ece35466-bib-0100] Partridge, L. , & Harvey, P. H. (1988). The ecological context of life history evolution. Science, 241, 1449–1455. 10.1126/science.241.4872.1449 17790040

[ece35466-bib-0101] Pinheiro, J. , Bates, D. , DebRoy, S. , Sarkar, D. , & R Development Core Team . (2016). nlme: Linear and nonlinear mixed effects models. R package version 3.1‐125. Retrieved from http://CRAN.R-project.org/package=nlme

[ece35466-bib-0102] Ploger, B. J. (1997). Does brood reduction provide nestling survivors with a food bonus? Animal Behaviour, 54, 1063–1076. 10.1006/anbe.1997.0503 9398363

[ece35466-bib-0103] Poole, A. (1982). Brood reduction in temperate and sub‐tropical ospreys. Oecologia, 53, 111–119. 10.1007/BF00377144 28310611

[ece35466-bib-0104] Prevost, Y. A. (1982). Wintering ecology of ospreys in Senegambia. PhD thesis, University of Edinburgh.

[ece35466-bib-0105] R Development Core Team (2018). R: A language and environment for statistical computing. Vienna, Austria: R Foundation for Statistical Computing Retrieved from: http://www.R-project.org/

[ece35466-bib-0106] Remeš, V. (2007). Avian growth and development rates and age‐specific mortality: The roles of nest predation and adult mortality. Journal of Evolutionary Biology, 20, 320–325. 10.1111/j.1420-9101.2006.01191.x 17210025

[ece35466-bib-0107] Remeš, V. , & Matysioková, B. (2016). Survival to independence in relation to pre‐fledging development and latitude in songbirds across the globe. Journal of Avian Biology, 47, 610–618. 10.1111/jav.00841

[ece35466-bib-0108] Reznick, D. , Bryant, M. J. , & Bashey, F. (2002). r‐and K‐selection revisited: The role of population regulation in life‐history evolution. Ecology, 83, 1509–1520. 10.2307/3071970

[ece35466-bib-0109] Ricklefs, R. E. (2000). Density dependence, evolutionary optimization, and the diversification of avian life histories. The Condor, 102, 9–22. 10.1650/0010-5422(2000)102[0009:DDEOAT]2.0.CO;2

[ece35466-bib-0110] Ricklefs, R. E. (2010). Parental investment and avian reproductive rate: Williams's principle reconsidered. The American Naturalist, 175, 350–361. 10.1086/650371 20109060

[ece35466-bib-0111] Romero, J. M. , & Redondo, T. (2017). Kind to kin: Weak interference competition among white stork Ciconia ciconia broodmates. Journal of Avian Biology, 48, 417–430. 10.1111/jav.00983

[ece35466-bib-0112] Rotics, S. , Kaatz, M. , Resheff, Y. S. , Turjeman, S. F. , Zurell, D. , Sapir, N. , … Nathan, R. (2016). The challenges of the first migration: Movement and behaviour of juvenile vs. adult white storks with insights regarding juvenile mortality. Journal of Animal Ecology, 85, 938–947. 10.1111/1365-2656.12525 27046512

[ece35466-bib-0113] Roulin, A. , & Wink, M. (2004). Predator‐prey relationships and the evolution of colour polymorphism: A comparative analysis in diurnal raptors. Biological Journal of the Linnean Society, 81, 565–578. 10.1111/j.1095-8312.2004.00308.x

[ece35466-bib-0114] Rutz, C. (2012). Predator fitness increases with selectivity for odd prey. Current Biology, 22, 820–824. 10.1016/j.cub.2012.03.028 22503502

[ece35466-bib-0115] Rutz, C. , Whittingham, M. J. , & Newton, I. (2006). Age‐dependent diet choice in an avian top predator. Proceedings of the Royal Society of London B: Biological Sciences, 273, 579–586. 10.1098/rspb.2005.3353 PMC1560053 16537129

[ece35466-bib-0116] Sæther, B. E. (1994). Food provisioning in relation to reproductive strategy in altricial birds: A comparison of two hypotheses. Evolution, 48, 1397–1406. 10.1111/j.1558-5646.1994.tb05324.x 28564472

[ece35466-bib-0117] Sæther, B.‐E. , Coulson, T. , Grøtan, V. , Engen, S. , Altwegg, R. , Armitage, K. B. , … Weimerskirch, H. (2013). How life history influences population dynamics in fluctuating environments. The American Naturalist, 182, 743–759. 10.1086/673497 24231536

[ece35466-bib-0118] Schoener, T. W. (1968). Sizes of feeding territories among birds. Ecology, 49, 123–141. 10.2307/1933567

[ece35466-bib-0119] Schwagmeyer, P. L. , & Mock, D. W. (2008). Parental provisioning and offspring fitness: Size matters. Animal Behaviour, 75, 291–298. 10.1016/j.anbehav.2007.05.023

[ece35466-bib-0120] Sergio, F. , Tanferna, A. , De Stephanis, R. , Jiménez, L. L. , Blas, J. , Tavecchia, G. , … Hiraldo, F. (2014). Individual improvements and selective mortality shape lifelong migratory performance. Nature, 515, 410–413. 10.1038/nature13696 25252973

[ece35466-bib-0121] Sibly, R. M. , & Brown, J. H. (2007). Effects of body size and lifestyle on evolution of mammal life histories. Proceedings of the National Academy of Sciences of the United States of America, 104, 17707–17712. 10.1073/pnas.0707725104 17940028 PMC2077039

[ece35466-bib-0122] Sibly, R. M. , Witt, C. C. , Wright, N. A. , Venditti, C. , Jetz, W. , & Brown, J. H. (2012). Energetics, lifestyle, and reproduction in birds. Proceedings of the National Academy of Sciences of the United States of America, 109, 10937–10941. 10.1073/pnas.1206512109 22615391 PMC3390878

[ece35466-bib-0123] Simmons, R. E. (1988). Offspring quality and the evolution of cainism. Ibis, 130, 339–357. 10.1111/j.1474-919X.1988.tb08809.x

[ece35466-bib-0124] Simmons, R. E. (1991). Offspring quality and sibling aggression in the black eagle. Ostrich, 62, 89–92. 10.1080/00306525.1991.9639644

[ece35466-bib-0125] Simmons, R. E. (1993). Effects of supplementary food on density‐reduced breeding in an African eagle: Adaptive restraint or ecological constraint? Ibis, 135, 394–402. 10.1111/j.1474-919X.1993.tb02111.x

[ece35466-bib-0126] Simmons, R. E. (2000). Harriers of the world: Their behaviour and ecology. New York, NY: Oxford University Press.

[ece35466-bib-0127] Simmons, R. E. (2002). Siblicide provides food benefits for raptor chicks: Re‐evaluating brood manipulation studies. Animal Behaviour, 64, 19–24. 10.1006/anbe.2002.2021

[ece35466-bib-0128] Smith, K. G. , Wittenberg, S. R. , Macwhirter, R. B. , & Bildstein, K. L. (2011). Hen/Northern Harrier (*Circus cyaneus/hudsonius*), version 2.0 In Poole A. F., & Gill F. B. (Eds.), The birds of North America. Ithaca, NY: The Cornell Lab of Ornithology 10.2173/bna.210

[ece35466-bib-0129] Squires, J. R. , & Reynolds, R. T. (1997). Northern goshawk (*Accipiter gentilis*), version 2.0 In Poole A. F., & Gill F. B. (Eds.), The birds of North America. Ithaca, NY: The Cornell Lab of Ornithology 10.2173/bna.298

[ece35466-bib-0130] Starck, J. M. , & Ricklefs, R. E. (1998). Avian growth and development: Evolution within the altricial‐precocial spectrum. New York, NY: Oxford University Press.

[ece35466-bib-0131] Stearns, S. C. (1976). Life‐history tactics: A review of the ideas. The Quarterly Review of Biology, 51, 3–47. 10.1086/409052 778893

[ece35466-bib-0132] Stinson, C. H. (1979). On the selective advantage of fratricide in raptors. Evolution, 33, 1219–1225. 10.1111/j.1558-5646.1979.tb04775.x 28563923

[ece35466-bib-0133] Sullivan, K. A. (1989). Predation and starvation: Age‐specific mortality in juvenile juncos (*Junco phaenotus*). Journal of Animal Ecology, 58, 275–286. 10.2307/5000

[ece35466-bib-0134] Symonds, M. R. , & Blomberg, S. P. (2014). A primer on phylogenetic generalised least squares In Garamszegi L. Z. (Ed.), Modern phylogenetic comparative methods and their application in evolutionary biology (pp. 105–130). Berlin, Germany: Springer.

[ece35466-bib-0135] Symonds, M. R. , & Moussalli, A. (2011). A brief guide to model selection, multimodel inference and model averaging in behavioural ecology using Akaike's information criterion. Behavioral Ecology and Sociobiology, 65, 13–21. 10.1007/s00265-010-1037-6

[ece35466-bib-0136] Tarwater, C. E. , Ricklefs, R. E. , Maddox, J. D. , & Brawn, J. D. (2011). Pre‐reproductive survival in a tropical bird and its implications for avian life histories. Ecology, 92, 1271–1281. 10.1890/10-1386.1 21797155

[ece35466-bib-0137] Temeles, E. J. (1985). Sexual size dimorphism of bird‐eating hawks: The effect of prey vulnerability. The American Naturalist, 125, 485–499. 10.1086/284357

[ece35466-bib-0138] Terraube, J. , Arroyo, B. , Madders, M. , & Mougeot, F. (2011). Diet specialisation and foraging efficiency under fluctuating vole abundance: A comparison between generalist and specialist avian predators. Oikos, 120, 234–244. 10.1111/j.1600-0706.2010.18554.x

[ece35466-bib-0139] Toland, B. (1986). Hunting success of some Missouri raptors. Wilson Bulletin, 98, 116–125.

[ece35466-bib-0140] Turner, D. P. , Ritts, W. D. , Cohen, W. B. , Gower, S. T. , Running, S. W. , Zhao, M. , … Ahl, D. E. (2006). Evaluation of MODIS NPP and GPP products across multiple biomes. Remote Sensing of Environment, 102, 282–292. 10.1016/j.rse.2006.02.017

[ece35466-bib-0141] Varland, D. E. , Klass, E. E. , & Loughin, T. M. (1991). Development of foraging behavior in the American kestrel. Journal of Raptor Research, 25, 9–17.

[ece35466-bib-0142] Viñuela, J. (1999). Sibling aggression, hatching asynchrony, and nestling mortality in the black kite (*Milvus migrans*). Behavioral Ecology and Sociobiology, 45, 33–45. 10.1007/s002650050537

[ece35466-bib-0143] von Hardenberg, A. , & González‐Voyer, A. (2013). Disentagling evolutionary cause‐effect relationships with phylogenetic confirmatory path analysis. Evolution, 67, 378–387. 10.1111/j.1558-5646.2012.01790.x 23356611

[ece35466-bib-0144] von Schantz, T. , Nilsson, I. N. , & von Schantz, T. (1981). The reversed size dimorphism in birds of prey: A new hypothesis. Oikos, 36, 129–132. 10.2307/3544388

[ece35466-bib-0145] Warnes, G. R. , Bolker, B. , & Lumley, T. (2018). gtools: Various R Programming Tools. R package version 3.8.1. Retrieved from https://CRAN.R-project.org/package=gtools

[ece35466-bib-0146] Warton, D. I. , & Hui, F. K. (2011). The arcsine is asinine: The analysis of proportions in ecology. Ecology, 92, 3–10. 10.1890/10-0340.1 21560670

[ece35466-bib-0147] Watson, R. T. , Razafindramanana, S. , Thorstrom, R. , & Rafanomezantsoa, S. (1999). Breeding biology, extra‐pair birds, productivity, siblicide and conservation of the Madagascar fish eagle. Ostrich, 70, 105–111. 10.1080/00306525.1999.9634522

[ece35466-bib-0148] Western, D. , & Ssemakula, J. (1982). Life history patterns in birds and mammals and their evolutionary interpretation. Oecologia, 54, 281–290. 10.1007/BF00379994 28309949

[ece35466-bib-0149] Wiens, J. D. , Noon, B. R. , & Reynolds, R. T. (2006). Post‐fledging survival of northern goshawks: The importance of prey abundance, weather, and dispersal. Ecological Applications, 16, 406–418. 10.1890/04-1915 16705989

[ece35466-bib-0150] Wilbur, H. M. , Tinkle, D. W. , & Collins, J. P. (1974). Environmental certainty, trophic level, and resource availability in life history evolution. The American Naturalist, 108, 805–817. 10.1086/282956

[ece35466-bib-0151] Wink, M. , & Sauer‐Gürth, H. (2004). Phylogenetic relationships in diurnal raptors based on nucleotide sequences of mitochondrial and nuclear marker genes In Chancelor R. D., & Meyburg B. U. (Eds.), Raptors worldwide (pp. 483–498). Berlin, Germany: WWGBP.

[ece35466-bib-0152] Zhang, Y. , Xu, M. , Chen, H. , & Adams, J. (2009). Global pattern of NPP to GPP ratio derived from MODIS data: Effects of ecosystem type, geographical location and climate. Global Ecology and Biogeography, 18, 280–290. 10.1111/j.1466-8238.2008.00442.x

[ece35466-bib-0153] Zhao, M. , Heinsch, F. A. , Nemani, R. R. , & Running, S. W. (2005). Improvements of the MODIS terrestrial gross and net primary production global data set. Remote Sensing of Environment, 95, 164–176. 10.1016/j.rse.2004.12.011

[ece35466-bib-0154] Zuur, A. F. , Ieno, E. N. , & Elphick, C. S. (2010). A protocol for data exploration to avoid common statistical problems. Methods in Ecology and Evolution, 1, 3–14. 10.1111/j.2041-210X.2009.00001.x

