# Supplementary material for: Broodmate aggression and life history variation in accipitrid birds of prey
Source: Ecol Evol. 2019 Jul 23;9(16):9185–206. doi: 10.1002/ece3.5466 (PMC6706193; doi:10.1002/ece3.5466)

Appendix S1. Testing potential biases due to heterogeneity in sampling effort

Table A1. The minimum number of broods observed for estimating behavioural variables

| Species | Intensity of aggression | Provisioning rate | Feeding method |
| --- | --- | --- | --- |
| *Accipiter badius* | 6 | 1 | 2 |
| *Accipiter cirrocephalus* | 2 | 3§ | NA |
| *Accipiter cooperii* | 11 | 13 | 2 |
| *Accipiter gentilis* | 17 | 22 | 3 |
| *Accipiter melanoleucus* | 3 | 1 | 1 |
| *Accipiter minullus* | 2 | 1 | 1 |
| *Accipiter nisus* | 50 | 7 | 3 |
| *Accipiter novaehollandiae* | 27 | 3§ | 3 |
| *Accipiter rufiventris* | 2 | 1 | 2 |
| *Accipiter soloensis* | 8 | 8 | 8 |
| *Accipiter striatus* | 4 | 1 | 5 |
| *Aquila adalberti* | 14 | 7 | NA |
| *Aquila audax* | 20 | 2 | 1 |
| *Aquila chrysaetos* | 13 | 4 | 11 |
| *Aquila clanga* | 2 | 1 | NA |
| *Aquila fasciatus* | 50 | 8 | 1 |
| *Aquila pomarina* | 10 | 5 | 10 |
| *Aquila verreauxii* | 40 | 1 | 39 |
| *Aquila wahlbergi* | 8 | 4 | 3 |
| *Aviceda subcristata* | 7 | 4 | 7 |
| *Butastur rufipennis* | 29 | 26 | 29 |
| *Buteo albonotatus* | 2 | 5 | 12 |
| *Buteo augur* | 3 | 8 | NA |
| *Buteo brachyurus* | 2 | 3 | 1 |
| *Buteo buteo* | 3 | 4 | 5 |
| *Buteo galapagoensis* | 10 | 4 | NA |
| *Buteo jamaicensis* | 2 | 1 | 50 |
| *Buteo lagopus* | 344 | 1 | 344 |
| *Buteo lineatus* | 4 | 4 | 21 |
| *Buteo platypterus* | 10 | 5 | 4 |
| *Buteo regalis* | 36 | 12 | 36 |
| *Buteo ridgwayi* | 3 | 3 | NA |
| *Buteo swainsoni* | 4 | 14 | 3 |
| *Circus aeruginosus* | 2§ | 2 | 19 |
| *Circus approximans* | 4§ | 2§ | 2 |
| *Circus cyaneus* | 2 | 5 | 3 |
| *Circus pygargus* | 4§ | 7 | 3 |
| *Elanoides forficatus* | 9 | 21 | 22 |
| *Elanus axillaris* | 4 | 1 | 1 |
| *Elanus scriptus* | 20 | 1§ | 1 |
| *Geranoaetus melanoleucus* | 5 | 1 | NA |
| *Gypaetus barbatus* | 7 | 2 | 7 |
| *Haliaeetus albicilla* | 7§ | 7 | 5 |
| *Haliaeetus leucocephalus* | 5 | 1 | 10 |
| *Haliaeetus leucogaster* | 4 | 1 | 1 |
| *Haliaeetus vocifer* | 4 | 10 | 2 |
| *Haliaeetus vociferoides* | 10 | 1 | 10 |
| *Hamirostra melanosternon* | 2 | 1 | 1 |
| *Hieraaetus morphnoides* | 4 | 2 | 4 |
| *Hieraaetus pennatus* | 21 | 3 | 5 |
| *Hieraaetus spilogaster* | 2 | 2 | 2 |
| *Ictinia mississippiensis* | 2§ | 10 | 2 |
| *Lophaetus occipitalis* | 3 | 3 | NA |
| *Lophoictinia isura* | 2 | 2§ | 2 |
| *Melierax canorus* | 3 | 19 | 3 |
| *Milvus migrans* | 100 | 20 | 10 |
| *Milvus milvus* | 2§ | 1§ | 4 |
| *Neophron percnopterus* | 10 | 4 | 1 |
| *Pandion haliaetus* | 55 | 4 | 4 |
| *Parabuteo unicinctus* | 3 | 64 | 3 |
| *Pernis apivorus* | 47 | 3 | 47 |
| *Polyboroides typus* | 4 | 3 | 4 |
| *Rostrhamus sociabilis* | 20 | 18 | 20 |
| *Sagittarius serpentarius* | 5§ | 2 | 10 |
| *Stephanoaetus coronatus* | 3§ | 4 | 3 |

§ number of bibliographic sources

Testing potential biases due to heterogeneity in sampling effort

1) We computed effect sizes (as both phylogenetically adjusted and nonparametric correlation coefficients and their associated 95 % confidence intervals) to determine the strength and direction of each particular relationship between estimates of behavioural variables and the sample sizes from which they were obtained (Garamszegi & Møller, 2012). We found no evidence that heterogeneity in sampling effort introduced a systematic bias in estimates of intensity of broodmate aggression, i.e. species reported to be more aggressive were not better studied (Table A2). Similarly, no positive or negative correlation was detected between provisioning rate and sampling effort. A negative effect was however found for feeding method: species that were better studied tended to be estimated as feeding their chicks more indirectly.

Table A2. The relationship between three behavioural variables and the sample sizes (minimum number of broods observed) from which they were estimated. Pearson correlation effect sizes and the associated 95 % confidence intervals are based on phylogenetically adjusted correlations. Lambda values (λ) were first estimated based on maximum likelihood (sample sizes as predictors) and then used to compute covariances and Pearson correlations. Also given are Spearman correlation coefficients computed on ranked variables with no phylogenetic adjustment.

| Variable | λ | Pearson r | 95% CI | Spearman | 95% CI |
| --- | --- | --- | --- | --- | --- |
| aggression | 0.00 | 0.074 | -0.420 / 0.568 | 0.096 | -0.151 / 0.332 |
| provisioning rate | 0.85 | -0.108 | -0.600 / 0.384 | 0.068 | -0.179 / 0.307 |
| feeding method | 0.00 | -0.311 | -0.782 / 0.160 | -0.303 | -0.522 / -0.046 |

2) To assess the effect of heterogeneity in sampling effort among species, we ran both ordinary (non-adjusted) PGLS and PGLS adjusted for differences in the minimum number of broods ni among species by means of weighted regressions where 1/[log(1+ni)] was supplied as an estimate of within-species variance component (Garamszegi, 2014). The log transformation helps downweighting data points with very low sample sizes, but without making too much discrimination between species-specific trait estimates that come from a reasonably large within-species samples (Garamszegi & Møller, 2010). Next, we checked the model fit statistics (AIC) to verify whether weighted models accounting for within-species variance offered a better fit to the data. Invariably, we found that the best model is the phylogenetic model that does not consider weights. All weighted models overemphasized the influence of the difference in sample sizes (Table A3). Accordingly, we used non-weighted PGLS in comparative analyses.

Table A3. Comparison of regression coefficients (β) (±SE) and their associated P values, and model fit statistics (AIC) for ordinary PGLS models (unweighted) and models weighted by the inverse of the minimum number of broods observed (1/[log(1+n)]) for estimating the three behavioural variables: intensity of broodmate aggression (AG), nestling provisioning rate (PR), and feeding method (FM)

|  | Unweighted model | | | | Weighted model | | | | |
| --- | --- | --- | --- | --- | --- | --- | --- | --- | --- |
| Model | **β** | **SE** | **P** | **AIC** | **Weights** | **β** | **SE** | **P** | **AIC** |
| aggression ~ provisioning rate | -0.62 | 0.15 | <0.001 | 201.2 | n AG | -0.96 | 0.10 | <0.001 | 228.2 |
| aggression ~ clutch size | -1.70 | 0.43 | <0.001 | 202.8 | n AG | -2.92 | 0.38 | <0.001 | 242.6 |
| aggression ~ body mass | 0.46 | 0.15 | 0.003 | 208.1 | n AG | 0.59 | 0.22 | 0.010 | 275.7 |
| aggression ~ nestling period | 1.12 | 0.40 | 0.007 | 209.9 | n AG | 2.06 | 0.52 | <0.001 | 270.7 |
| aggression ~ feeding method | -0.12 | 0.08 | 0.177 | 188.8 | n AG | 0.26 | 0.10 | 0.013 | 242.6 |
| provisioning rate ~ aggression | -0.26 | 0.08 | 0.001 | 156.3 | n PR | -0.24 | 0.09 | 0.011 | 207.4 |
| feeding method ~ aggression | -0.27 | 0.20 | 0.178 | 236.3 | n FM | 0.27 | 0.13 | 0.052 | 247.8 |

3) We tested whether sampling effort showed a phylogenetic signal (λ>0) by computing maximum likelihood profiles of λ values in null PGLS models with minimum number of broods for each behavioural variable as the response variable (Garamszegi & Mundry, 2014). We detected no evidence for sampling effort being structured by phylogenetic relatedness, i.e. there is no evident trend in our sample for certain taxa being better studied than others (Garamszegi & Møller, 2012) (Figure A1).

Figure A1. Maximum likelihood surfaces of the phylogenetic scaling factor lambda (λ) for estimating evidence of phylogenetic signal in the sampling effort (number of nests) data for the three behavioural variables in the study. Upper: intensity of broodmate aggression; Middle: provisioning rate; Lower: feeding method.


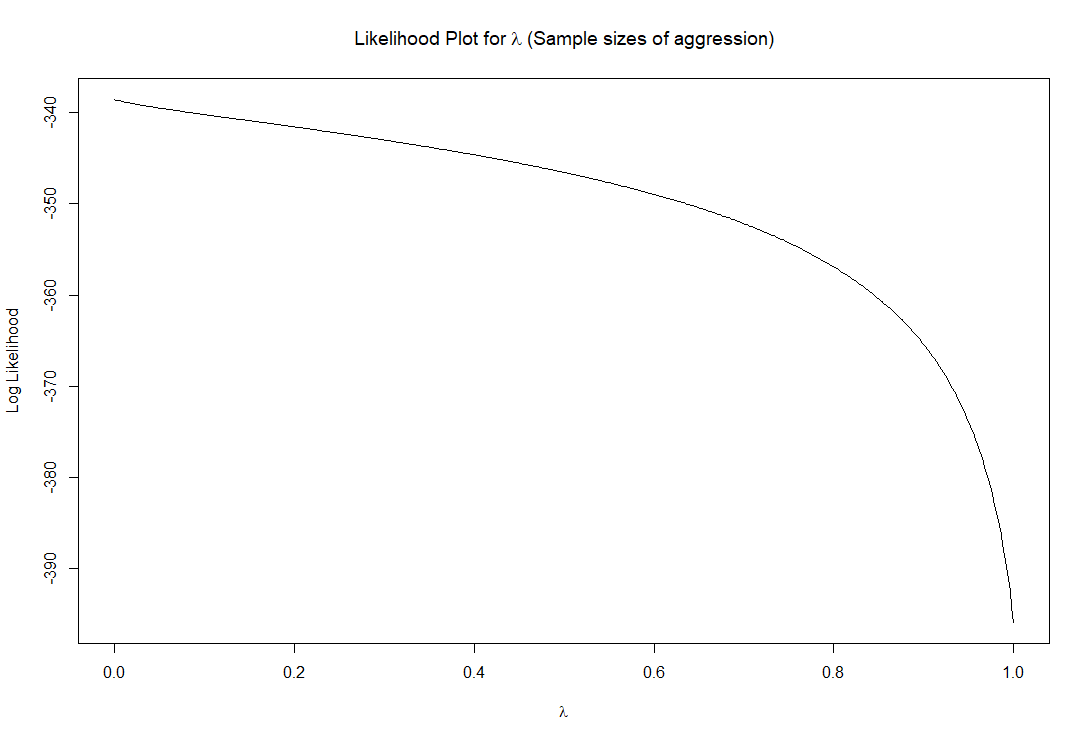


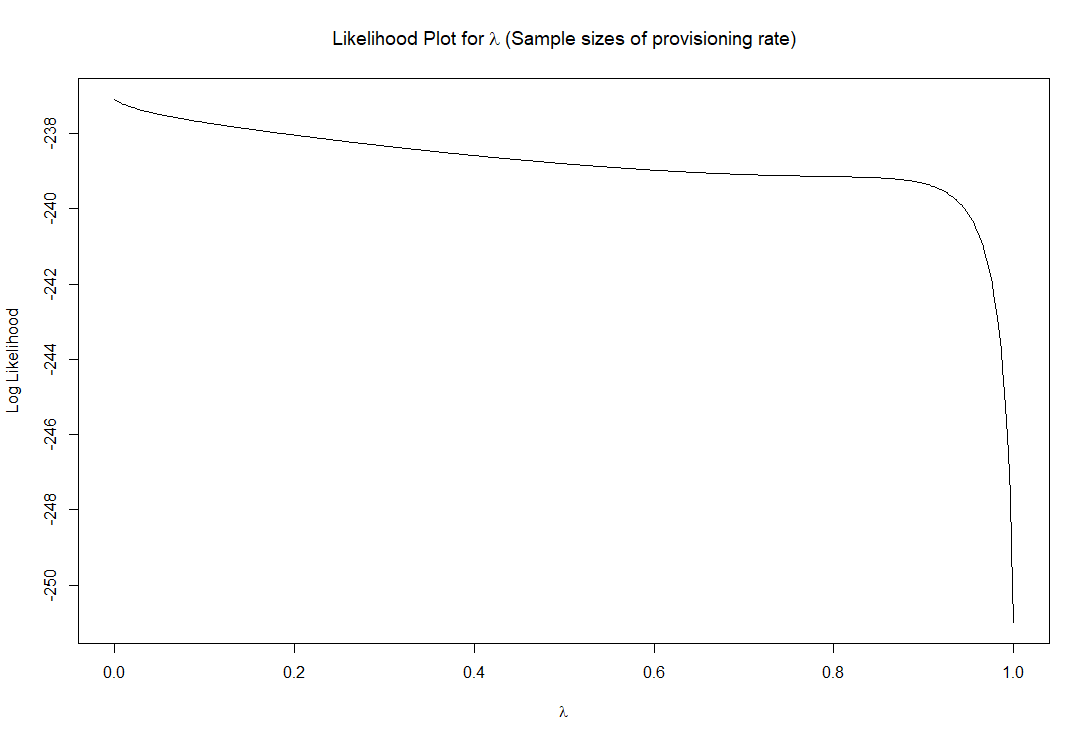


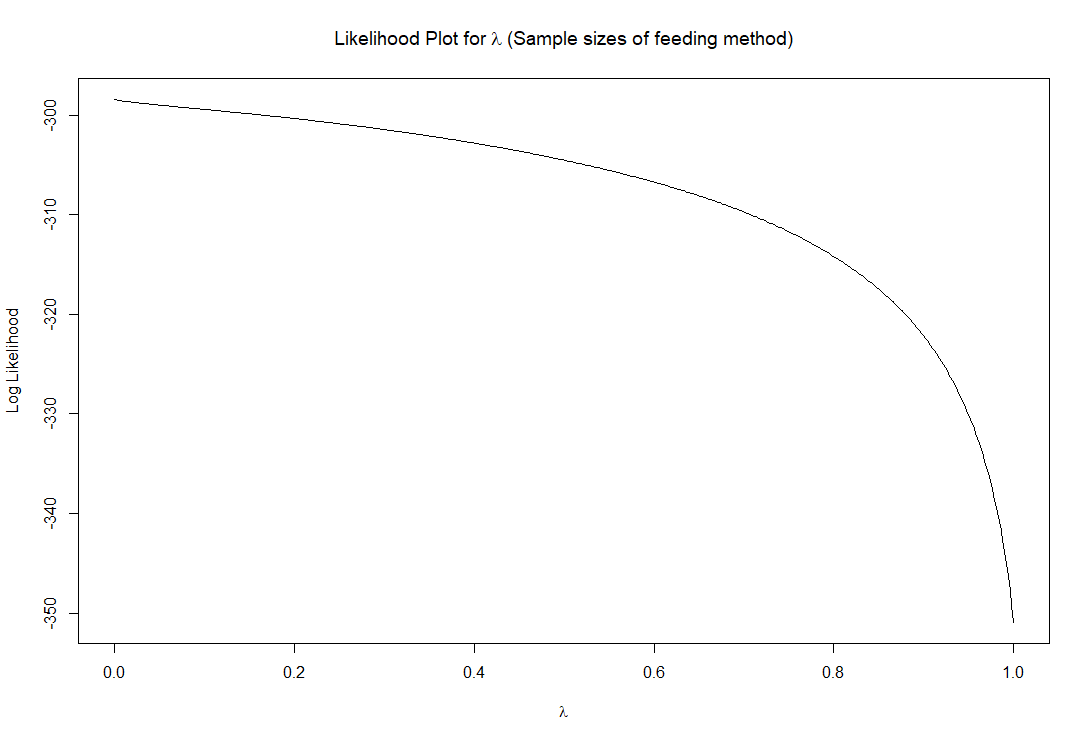

Supplement: Supplementary file 1 [file ECE3-9-9185-s001.docx]
