# Supplementary material for: Broodmate aggression and life history variation in accipitrid birds of prey
Source: Ecol Evol. 2019 Jul 23;9(16):9185–206. doi: 10.1002/ece3.5466 (PMC6706193; doi:10.1002/ece3.5466)
Supplement: Supplementary file 2 [file ECE3-9-9185-s002.docx]

Appendix S2. Design of evolutionary causal layouts used in Path Analysis

The design of path analysis started by defining three basic evolutionary layouts, which differed in the root variable which was hypothesized to act as the primary cause of all other variables: PR (Layout X), BM (Layout Y) or CS (Layout Z) which correspond to the three evolutionary scenarios in Figure 2. In each layout, the root variable defines a set of primary causal relationships (edges), i.e. those connecting the root variable as a causal predictor and all other traits (except AG) as causal dependent variables, with seven possible combinations of primary edges for each layout (Table B1).

As a second step, we defined a set of secondary causal relationships, i.e. those existing among variables other than the root variable (and AG), amounting 12 possible combinations of secondary edges for each layout (Table B1). At this point, we assumed that all non-rooted variables except AG might act either as causally dependent or independent, on the basis of plausible, biologically meaningful mechanisms (see below), with one exception: NP is never a causal predictor of either BM, PR or CS (e.g. Martin, 1995).

Finally, for every layout, there are seven possible combinations of tertiary edges defining the direct causal relationships between CS, NP and PR (as causal predictors) and AG (as the causally dependent variable) (Table B1).

Table B1. Possible combinations of direct causal relationships between pairs of variables defining the space of model paths. Causal relationships are classified as Primary (those between the root variable defining each layout and all other variables except Aggression (AG), Secondary (those between pairs of variables other than the root variable and AG), and Tertiary (those between all variables and AG. Body mass (BM) is assumed to be a direct cause of either clutch size (CS), nestling period (NP), or provisioning rate (PR), but not AG.

|  | Primary |  | Secondary |  | Tertiary |
| --- | --- | --- | --- | --- | --- |
|  |  |  |  |  |  |
| Layout X: PR is the root variable | | | | | |
| 1 | PR→BM, PR→CS, PR→NP | | BM→CS, BM→NP, CS→NP | | CS→AG, PR→AG, NP→AG |
| 2 | PR→CS, PR→NP | | CS→BM, BM→NP, CS→NP | | CS→AG, PR→AG |
| 3 | PR→BM, PR→NP | | BM→CS, BM→NP | | CS→AG, NP→AG |
| 4 | PR→BM, PR→CS | | CS→BM, BM→NP | | PR→AG, NP→AG |
| 5 | PR→BM |  | BM→CS, CS→NP | | CS→AG |
| 6 | PR→CS |  | CS→BM, CS→NP | | PR→AG |
| 7 | PR→NP |  | BM→NP, CS→NP | | NP→AG |
| 8 |  |  | BM→CS |  |  |
| 9 |  |  | CS→BM |  |  |
| 10 |  |  | BM→NP |  |  |
| 11 |  |  | CS→NP |  |  |
| 12 |  |  | Ø |  |  |
|  |  |  |  |  |  |
| Layout Y: BM is the root variable | | | | | |
| 1 | BM→CS, BM→PR, BM→NP | | PR→CS, PR→NP, CS→NP | | CS→AG, PR→AG, NP→AG |
| 2 | BM→PR, BM→NP | | CS→PR, PR→NP, CS→NP | | CS→AG, PR→AG |
| 3 | BM→CS, BM→NP | | PR→CS, PR→NP | | CS→AG, NP→AG |
| 4 | BM→CS, BM→PR | | CS→PR, PR→NP | | PR→AG, NP→AG |
| 5 | BM→CS |  | PR→CS, CS→NP | | CS→AG |
| 6 | BM→PR |  | CS→PR, CS→NP | | PR→AG |
| 7 | BM→NP |  | PR→NP, CS→NP | | NP→AG |
| 8 |  |  | PR→CS |  |  |
| 9 |  |  | CS→PR |  |  |
| 10 |  |  | PR→NP |  |  |
| 11 |  |  | CS→NP |  |  |
| 12 |  |  | Ø |  |  |
|  |  |  |  |  |  |
| Layout Z: CS is the root variable | | | | | |
| 1 | CS→BM, CS→PR, CS→NP | | BM→PR, BM→NP, PR→NP | | CS→AG, PR→AG, NP→AG |
| 2 | CS→PR, CS→NP | | PR→BM, BM→NP, PR→NP | | CS→AG, PR→AG |
| 3 | CS→BM, CS→NP | | BM→PR, BM→NP | | CS→AG, NP→AG |
| 4 | CS→BM, CS→PR | | PR→BM, BM→NP | | PR→AG, NP→AG |
| 5 | CS→BM |  | BM→PR, PR→NP | | CS→AG |
| 6 | CS→PR |  | PR→BM, PR→NP | | PR→AG |
| 7 | CS→NP |  | BM→NP, PR→NP | | NP→AG |
| 8 |  |  | PR→NP |  |  |
| 9 |  |  | BM→NP |  |  |
| 10 |  |  | PR→BM |  |  |
| 11 |  |  | BM→PR |  |  |
| 12 |  |  | Ø |  |  |

Each path model is defined by a unique combination of primary, secondary, and tertiary edges and named after it. For example, path Xp3s11t4 stands for a path defined by the third primary (PR →BM, PR→NP), eleventh secondary (CS→NP), and forth tertiary (PR→AG, NP→AG) combinations of edges corresponding to the basic Layout X (Table B1). A total of 1764 paths were generated by combining primary, secondary and tertiary edge combinations, of which 168 were unsolvable (e.g. they included cyclic, two-way or insufficient causal relationships). Also note that a given path can be generated by more than one layout, depending on the combination of edges chosen. We used an algorithm to identify duplicate paths, removing a total of 371 redundant paths. The final candidate model set consisted of 1225 solvable, unique paths (included as part of the dataset). As a result of removing redundant paths, path nomenclature in Table B1 does not correspond exactly to the evolutionary scenarios in Figure 2. For example, although both the Allometry scenario and Layout Y have in common that they assume BM is the root variable, model path Yp2s2t2 actually corresponds to the Fecundity evolutionary scenario because neither BM nor PR act as causal predictors of CS, while CS causes both PR and NP.
