# Supplementary material for: Broodmate aggression and life history variation in accipitrid birds of prey
Source: Ecol Evol. 2019 Jul 23;9(16):9185–206. doi: 10.1002/ece3.5466 (PMC6706193; doi:10.1002/ece3.5466)
Supplement: Supplementary file 3 [file ECE3-9-9185-s003.docx]

Appendix S3. Model selection table for behavioural and life-history predictors (excluding feeding method)

Table C1. Comparison of multiple regression models of intensity of aggression (response variable) and their life history predictors (excluding feeding method) when controlling for phylogeny using PGLS, ordered by AICc values. N = 65 species.

| Predictors | K | AICc | ΔAICc | L(g/data) | w | w' |
| --- | --- | --- | --- | --- | --- | --- |
| clutch size + provisioning rate | 4 | 170.62 | 0.000 | 1.000 | 0.403 | 0.694 |
| clutch size + provisioning rate + nestling period | 5 | 172.45 | 1.826 | 0.401 | 0.162 |  |
| clutch size + provisioning rate + body mass | 5 | 172.65 | 2.022 | 0.364 | 0.147 |  |
| provisioning rate | 3 | 173.58 | 2.952 | 0.229 | 0.092 | 0.159 |
| provisioning rate + body mass | 4 | 174.39 | 3.770 | 0.152 | 0.061 |  |
| clutch size + body mass | 4 | 175.18 | 4.559 | 0.102 | 0.041 | 0.071 |
| clutch size | 3 | 175.19 | 4.570 | 0.102 | 0.041 | 0.071 |
| provisioning rate + nestling period | 4 | 175.60 | 4.973 | 0.083 | 0.034 |  |
| clutch size + nestling period | 4 | 177.24 | 6.612 | 0.037 | 0.015 |  |
| body mass | 3 | 180.51 | 9.883 | 0.007 | 0.003 | 0.005 |
| nestling period | 3 | 182.33 | 11.703 | 0.003 | 0.001 |  |
| nestling period + body mass | 4 | 182.68 | 12.057 | 0.002 | 0.001 |  |
| Null | 2 | 186.93 | 16.303 | 0.000 | 0.000 |  |

K, number of parameters; AICc, Akaike’s information criterion with correction for small sample sizes; L(g/data), relative likelihood of a model given the data; w, probability (weight) of each model given the data and the set of models being compared; w’, weight of each model recalculated after excluding non truly-competitive models.
